# IGSF3 drives acquired temozolomide resistance in glioblastoma through ASNS-dependent asparagine biosynthesis

**DOI:** 10.1016/j.jbc.2026.113359

**Published:** 2026-07-22

**Authors:** Jie Li, Hongnian Lu, Qianqian Xu, Ying Yin, Zhening Pu, Jin Huang, Jingjing Wang

**Affiliations:** 1 Department of Laboratory Medicine, The Affiliated Wuxi People's Hospital of Nanjing Medical University, Wuxi People's Hospital, Wuxi Medical Center of Nanjing Medical University, Wuxi, Jiangsu, China; 2 Department of Neurosurgery, The Affiliated Wuxi People’s Hospital of Nanjing Medical University, Wuxi People’s Hospital, Wuxi Medical Center of Nanjing Medical University, Wuxi, Jiangsu, China

**Keywords:** glioblastoma, TMZ resistance, IGSF3, ASNS, asparagine

## Abstract

Glioblastoma (GBM) is the most aggressive primary malignancy of the central nervous system, and acquired resistance to temozolomide (TMZ) is a major cause of tumor recurrence and poor clinical outcomes. However, the tumor-intrinsic drivers and metabolic adaptations underlying this process remain incompletely understood. In this study, we integrated transcriptomic data from established TMZ-sensitive and TMZ-resistant models with public bulk and single-nucleus sequencing datasets, and validated candidate genes using clinical specimens, cell-based drug-sensitivity assays, xenograft models, metabolic measurements, luciferase reporter assays, chromatin immunoprecipitation, and small-molecule binding assays. Immunoglobulin superfamily member 3 (IGSF3) was consistently upregulated in TMZ-resistant cells, resistant xenografts, and recurrent GBM samples, and was mainly enriched in malignant glioma cells. Functionally, *IGSF3* overexpression promoted resistance to TMZ-related treatment, whereas *IGSF3* knockdown restored drug sensitivity. Mechanistically, IGSF3 was detected in the nucleus, where it enhanced the activity of the asparagine synthetase (ASNS) promoter. In addition, IGSF3 was found to be enriched at the *ASNS* promoter, thereby increasing ASNS expression and asparagine production, reduced reactive oxygen species accumulation, preserved glutathione redox balance, and limited DNA damage induced by treatment. ASNS gain- and loss-of-function rescue experiments showed that ASNS was required for the pro-resistance effect of IGSF3. Finally, Tucatinib bound to IGSF3, suppressed the IGSF3-ASNS pathway, and enhanced TMZ efficacy *in vitro* and *in vivo*. These findings identify the IGSF3-ASNS axis as a tumor-intrinsic metabolic adaptation that drives acquired TMZ resistance in GBM and suggest a potential therapeutic strategy for recurrent GBM.

Glioblastoma (GBM) is the most aggressive primary malignancy of the central nervous system (1, 2). Temozolomide (TMZ), an oral alkylating agent that efficiently crosses the blood-brain barrier, remains the standard first-line chemotherapy for GBM patients (3). However, despite an initial therapeutic response, approximately 50% of patients eventually develop acquired resistance following treatment, which serves as a decisive driver of tumor recurrence and dismal prognosis (4, 5). Therefore, it is imperative to elucidate the molecular mechanisms contributing to the acquisition of TMZ resistance to develop more effective strategies for clinical intervention.

The cytotoxic effect of TMZ is primarily attributed to its ability to induce DNA methylation, leading to the formation of O^6^-methylguanine and subsequent lethal DNA double-strand breaks (6). Although O^6^-methylguanine-DNA methyltransferase (MGMT)-mediated DNA repair is a well-established mechanism of TMZ resistance, acquired resistance can also emerge in MGMT-deficient or MGMT-low GBM models, including U87-based acquired-resistance systems (7, 8). This phenomenon suggests the involvement of alternative, MGMT-independent pathways that orchestrate therapeutic adaptation.

The immunoglobulin superfamily (IGSF) represents one of the largest and most diverse groups of cell surface and soluble proteins, primarily mediating cell-cell recognition, adhesion, and signal transduction (9, 10). Dysregulation of various IGSF members has been increasingly implicated in tumor progression and the modulation of therapeutic responses across multiple malignancies (11, 12, 13, 14). Specifically, IGSF3, a member of this superfamily, has recently been identified as a key participant in glioma cell invasion and neural development (15, 16). However, while the role of IGSF3 in glioma progression is becoming evident, its role in acquired TMZ resistance has not been systematically investigated.

Abnormal metabolic states contribute to a variety of diseases, including cancer (17, 18, 19). Asparagine synthetase (ASNS), the rate-limiting enzyme responsible for the biosynthesis of asparagine (Asn) from aspartate (Asp) and glutamine (Gln), plays a critical role in cellular adaptation to nutrient deprivation and therapeutic stress (20, 21, 22). High expression of ASNS has been correlated with tumor progression, biological aggressiveness, and chemoresistance in several malignancies (23, 24, 25). Moreover, by sustaining metabolic homeostasis and adaptive nutrient utilization, ASNS may enhance glioma stem cell survival under radio-therapy stress (26). However, the specific mechanisms by which ASNS-mediated metabolic shifts are associated with IGSF3 to drive acquired TMZ resistance remain to be determined.

In the present study, we systematically investigated the functional significance of IGSF3 in driving acquired TMZ resistance using our established mouse models. We demonstrate that IGSF3 is upregulated in recurrent and resistant GBM and promotes TMZ resistance through ASNS-dependent metabolic adaptation. Mechanistically, nuclear-localized IGSF3 transcriptionally activates ASNS by directly binding its promoter, leading to increased Asn production, improved oxidative stress buffering and reduced treatment-induced γH2AX accumulation. Furthermore, through virtual screening and experimental validation, we identified the FDA-approved drug Tucatinib as a candidate small-molecule modulator of the IGSF3-ASNS axis that enhances the antitumor efficacy of TMZ *in vitro* and *in vivo*. Our findings reveal a novel IGSF3-ASNS metabolic axis as a key driver of acquired TMZ resistance and provide a potential strategy to overcome chemoresistance in recurrent GBM.

## Results

### Integrated transcriptomic screening identifies differentially expressed genes (DEGs) associated with acquired TMZ resistance

**Figure 1:**
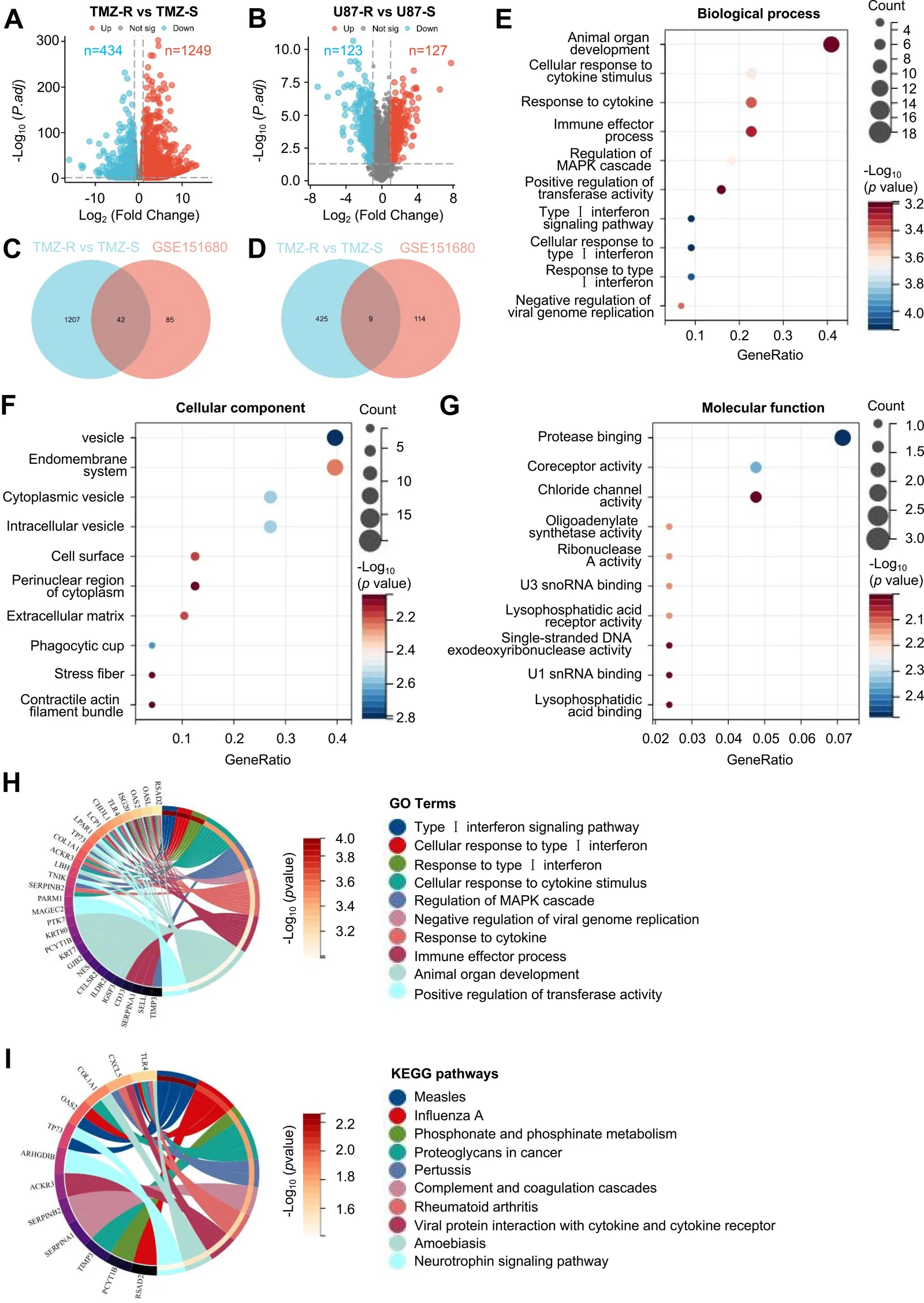
**Identification and functional enrichment analysis of conserved DEGs in acquired TMZ-resistant GBM models.***A* and *B*, volcano plots illustrating differentially expressed genes (DEGs) in the in-house U87 TMZ-resistant (TMZ-R) model *versus* sensitive (TMZ-S) model (*A*), and the GSE151680 dataset (*B*). *Red dots* represent upregulated genes, while *blue dots* represent downregulated genes. *C* and *D*, Venn diagrams showing the intersection of upregulated (*C*) and downregulated (*D*) DEGs between the two independent datasets. *E*, bubble diagram illustrating the enriched biological processes in Gene Ontology (GO) analysis. *F*, bubble diagram depicting the enriched cellular component in GO enrichment analysis. *G*, bubble diagram representing the molecular function in GO enrichment analysis. *H*, chord plot visualizing the specific DEGs in GO analysis. *I*, chord plot displaying the enriched KEGG pathways for the 51 common DEGs. DEG, differentially expressed gene; KEGG, Kyoto Encyclopedia of Genes and Genomes; TMZ, temozolomide.

To define tumor-intrinsic drivers of acquired TMZ resistance in GBM, we integrated transcriptomic data from our previously established *in vivo* TMZ-sensitive (TMZ-S) and TMZ-resistant (TMZ-R) U87 models with the public acquired TMZ-resistance dataset GSE151680. In our model, TMZ-S and TMZ-R xenograft tumors were designated as TMZ-S and TMZ-R, respectively, and the corresponding primary cells derived from these tumors were designated as U87-S and U87-R (27). We then performed differential expression analysis by comparing TMZ-R samples with their sensitive counterparts in each dataset. Differentially expressed genes (DEGs) were defined using the same cutoff criteria in both datasets: |log_2_FC| > 1.5 and *p* < 0.05. The volcano plots (Fig. 1, *A* and *B*) show substantial gene expression changes in both models. To focus on robust resistance-associated genes, we intersected the DEGs from the two datasets and retained only genes that showed consistent directional changes. This analysis defined a core set of 51 consistently altered genes, comprising 42 upregulated and nine downregulated genes (Fig. 1, *C* and *D*). These 51 common DEGs were selected for subsequent functional characterization.

To characterize the biological roles of these 51 common DEGs, we performed GO and KEGG pathway enrichment analyses. In the GO biological process category, the DEGs were significantly enriched for cellular responses to cytokine stimulus and immune effector processes (Fig. 1*E*). For CC category, the genes localized predominantly to vesicles, the endomembrane system, and the cell surface (Fig. 1*F*). In the molecular function category, the DEGs were enriched for protease binding and coreceptor activity (Fig. 1*G*). GO analysis further indicated involvement in type I interferon- and cytokine-driven immune responses, and in regulation of the MAPK cascade (Fig. 1*H*). KEGG enrichment highlighted pathways such as cytokine–cytokine receptor interaction, complement and coagulation cascades, and cancer-associated signaling including proteoglycans in cancer (Fig. 1*I*). Collectively, these findings indicate that acquired TMZ resistance in GBM is accompanied by coordinated remodeling of inflammatory signaling and cell surface components. We next focused on the consistently upregulated genes within this shared DEG set to identify candidate mediators that may actively contribute to the TMZ-R phenotype.

### Selection and clinical validation of IGSF3 as a key candidate in acquired TMZ resistance

**Figure 2:**
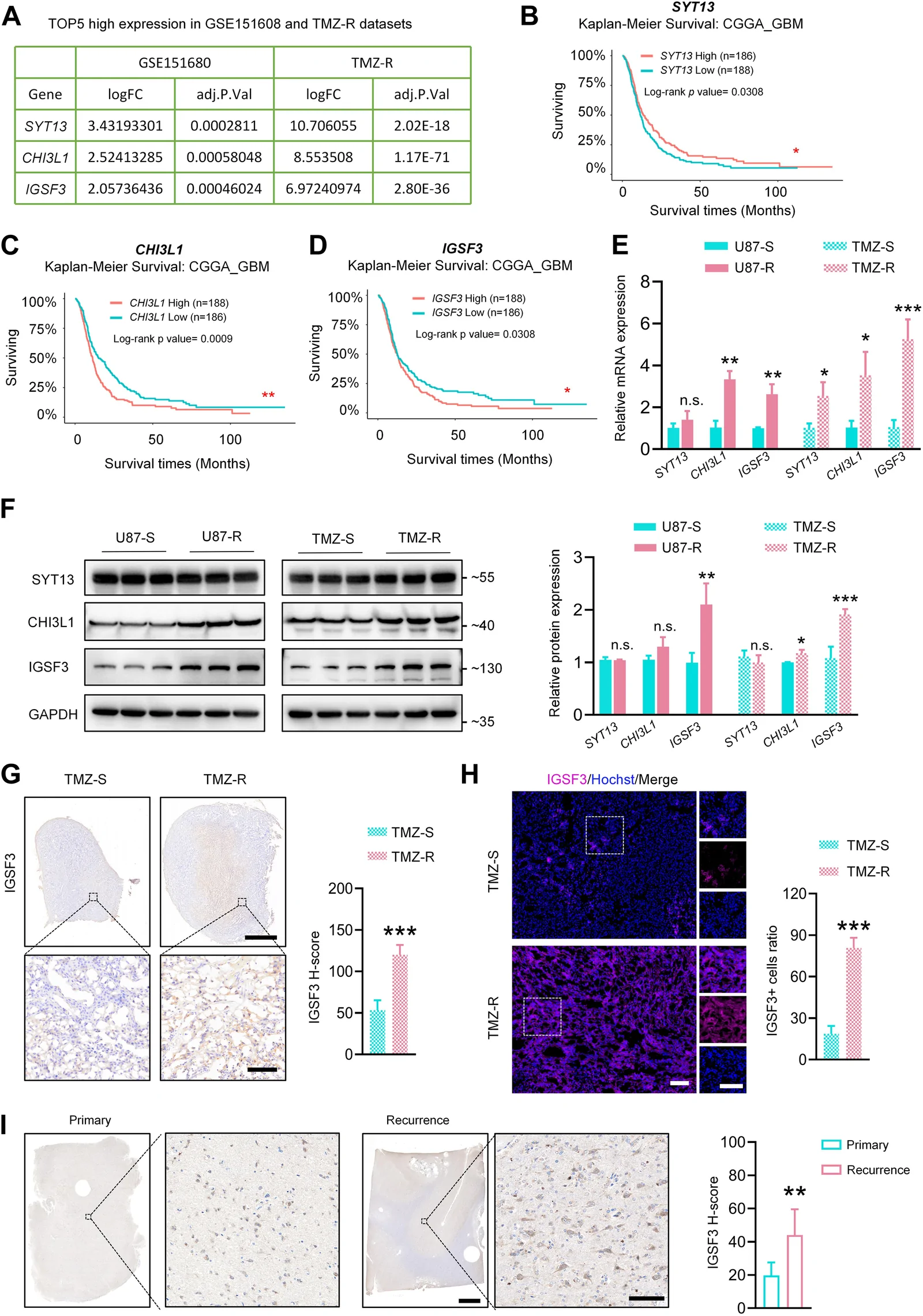
**Identification and validation of IGSF3 as a primary driver of TMZ resistance.***A*, candidate gene selection based on the rank of Log-fold change in our TMZ-R model and GSE151680 dataset. *B*, *C*, and *D*, Kaplan-Meier survival analysis of *SYT13* (*B*), *CHI3L1* (*C*), and *IGSF3* (*D*) in the CGGA GBM cohort (*n* = 374). Log-rank test *p*-values are indicated, ∗*p* < 0.05, ∗∗*p* < 0.01. *E*, *SYT13*, *CHI3L1* and *IGSF3* mRNA levels in TMZ-R mouse models, and U87-R cells (*n* = 3, ∗*p* < 0.05, ∗∗*p* < 0.01, ∗∗∗*p* < 0.001). *F*, representative image (*left*) and quantification (*right*) of SYT13, CHI3L1 and IGSF3 protein in tumor tissues and primary cells (*n* = 3, ∗*p* < 0.05, ∗∗*p* < 0.01, ∗∗∗*p* < 0.001). *G* representative immunohistochemistry (IHC) image (*left*) and H-score quantification (*right*) of IGSF3 in TMZ-S and TMZ-R xenograft tissues (the scale bars represent 2 mm (*upper*) and 100 μm (*lower*), ∗∗∗*p* < 0.001). *H*, representative immunofluorescence (IF) image (*left*) and quantification (*right*) of IGSF3 in TMZ-S and TMZ-R xenograft tissues (the scale bar represents 200 μm, ∗∗∗*p* < 0.001). *I*, representative IHC of primary (*n* = 8) and recurrent (*n* = 5) GBM tissues with representative IGSF3 staining (*left*) and H-score quantification (*right*) (the scale bars represent 2 mm (*left*) and 100 μm (*right*), ∗∗*p* < 0.01). CGGA, Chinese Glioma Genome Atlas; GBM, glioblastoma; IGSF, immunoglobulin superfamily; TMZ, temozolomide; TMZ-R, TMZ-resistant; TMZ-S, TMZ-sensitive.

Having defined a core set of resistance-associated genes, we next sought to identify candidates with both strong upregulation in resistant models and clinical relevance in GBM. To identify candidate mediators driving the TMZ-R phenotype, we prioritized the 42 upregulated genes by ranking their log-fold changes in both our TMZ-R and the GSE151680 datasets. This screening yielded three top-ranked candidates: *SYT13*, *CHI3L1*, and *IGSF3* (Fig. 2*A*). We then assessed their clinical relevance using Kaplan-Meier survival analysis in the CGGA GBM cohort. High expression of *CHI3L1* and *IGSF3* was significantly correlated with shorter overall survival, whereas high *SYT13* expression surprisingly associated with a more favorable prognosis (Fig. 2, *B*–*D*). To validate these findings, we examined the expression of these candidates in our TMZ-S and TMZ-R models. quantitative reverse transcription PCR (qRT-PCR) analysis demonstrated that all three candidates showed increased expression to different extents in resistant models, with *CHI3L1* and *IGSF3* displaying more evident mRNA upregulation. However, Western blot analysis showed that only IGSF3 was consistently and significantly elevated in both resistant cells and tumors, while CHI3L1 exhibited a weaker increase mainly in resistant tumor tissues and SYT13 showed no clear change at the protein level (Fig. 2, *E* and *F*). The high expression of IGSF3 in TMZ-R models was further corroborated by immunohistochemistry (IHC) and immunofluorescence (IF) staining (Fig. 2, *G* and *H*). These results further supported the prioritization of IGSF3 for subsequent investigation. Therefore, we extended our validation to clinical specimens from primary and recurrent GBM patients. Consistent with our model data, IHC staining revealed significantly higher IGSF3 protein levels in recurrent tumors compared with their primary counterparts (Fig. 2*I*). Collectively, these results identify IGSF3 as a robustly upregulated molecule in acquired TMZ resistance and a promising candidate for further functional investigation.

### Single-nucleus analysis reveals *IGSF3* enrichment in glioma cells and its association with tumor recurrence

**Figure 3:**
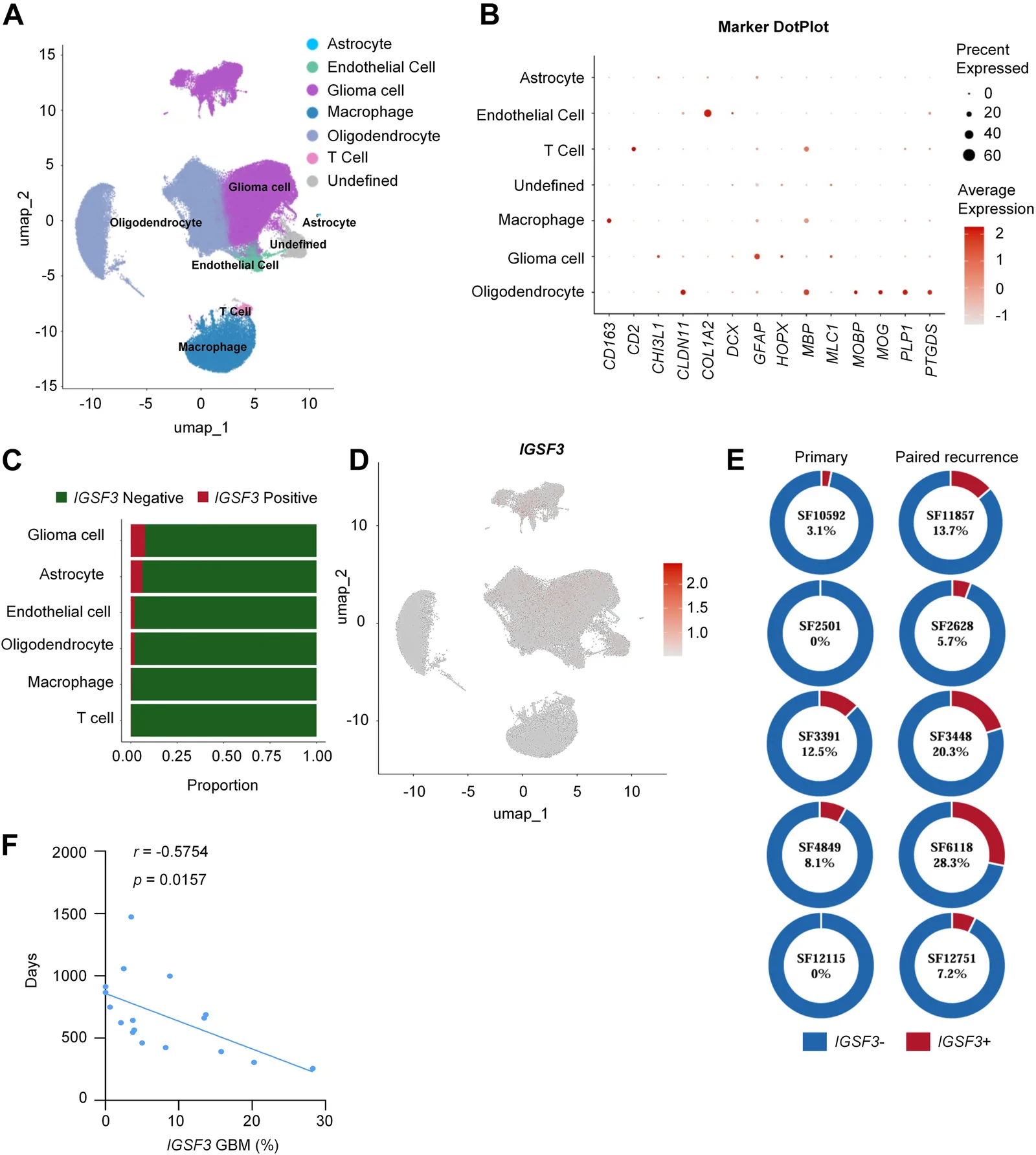
**Single-cell transcriptomic profiling of *IGSF3* in primary and recurrent GBM.***A*, UMAP visualization of cell clusters from the GSE174554 dataset. *B*, dot plot of marker genes used for cell type annotation. *C* and *D*, bar chart (*C*) and scatter plot (*D*) showing the cell-type-specific expression of *IGSF3*. *E*, representative quantification of *IGSF3*+ tumor cells in paired primary and recurrent GBM samples. *F*, Spearman correlation between the fraction of *IGSF3*+ tumor cells and overall survival. IGSF, immunoglobulin superfamily; UMAP, Uniform Manifold Approximation and projection.

Because bulk tumor analyses cannot distinguish whether IGSF3 upregulation originates from malignant cells or nonmalignant microenvironmental components, we next analyzed the GSE174554 single-nucleus RNA-seq dataset containing paired primary and recurrent GBM samples (Fig. 3*A*). Using canonical lineage markers (28), major cell populations were annotated, including astrocytes, endothelial cells, T cells, macrophages, oligodendrocytes, and malignant glioma cells (Fig. 3*B*).

Visualization of *IGSF3* expression across cell types revealed that *IGSF3* was predominantly expressed in malignant glioma cells (Fig. 3, *C* and *D*). This tumor-cell-restricted expression pattern suggests that IGSF3 upregulation observed in resistant tumors is primarily driven by intrinsic changes within glioma cells rather than microenvironmental components. We next compared the proportion of *IGSF3*-positive tumor cells between paired primary and recurrent lesions. Recurrent GBM samples exhibited a significantly higher fraction of *IGSF3*^+^ tumor cells than their primary counterparts (Fig. 3*E*), indicating enrichment of this subpopulation during tumor progression and therapeutic resistance. Importantly, correlation analysis demonstrated a significant negative association between the proportion of *IGSF3*^+^ tumor cells and patient overall survival (Fig. 3*F*), suggesting that expansion of the IGSF3-expressing tumor cell population is linked to adverse clinical outcome.

Collectively, these single-nucleus data indicate that IGSF3 is preferentially expressed in GBM cells and becomes enriched in recurrent tumors, supporting a functional role for *IGSF3*-positive tumor cell populations in GBM progression and acquired TMZ resistance.

### *IGSF3* modulates 5-(3-methyltriazen-1-yl)-imidazole-4-carboxamide (MTIC) resistance and the cellular response to DNA damage in GBM cells

**Figure 4:**
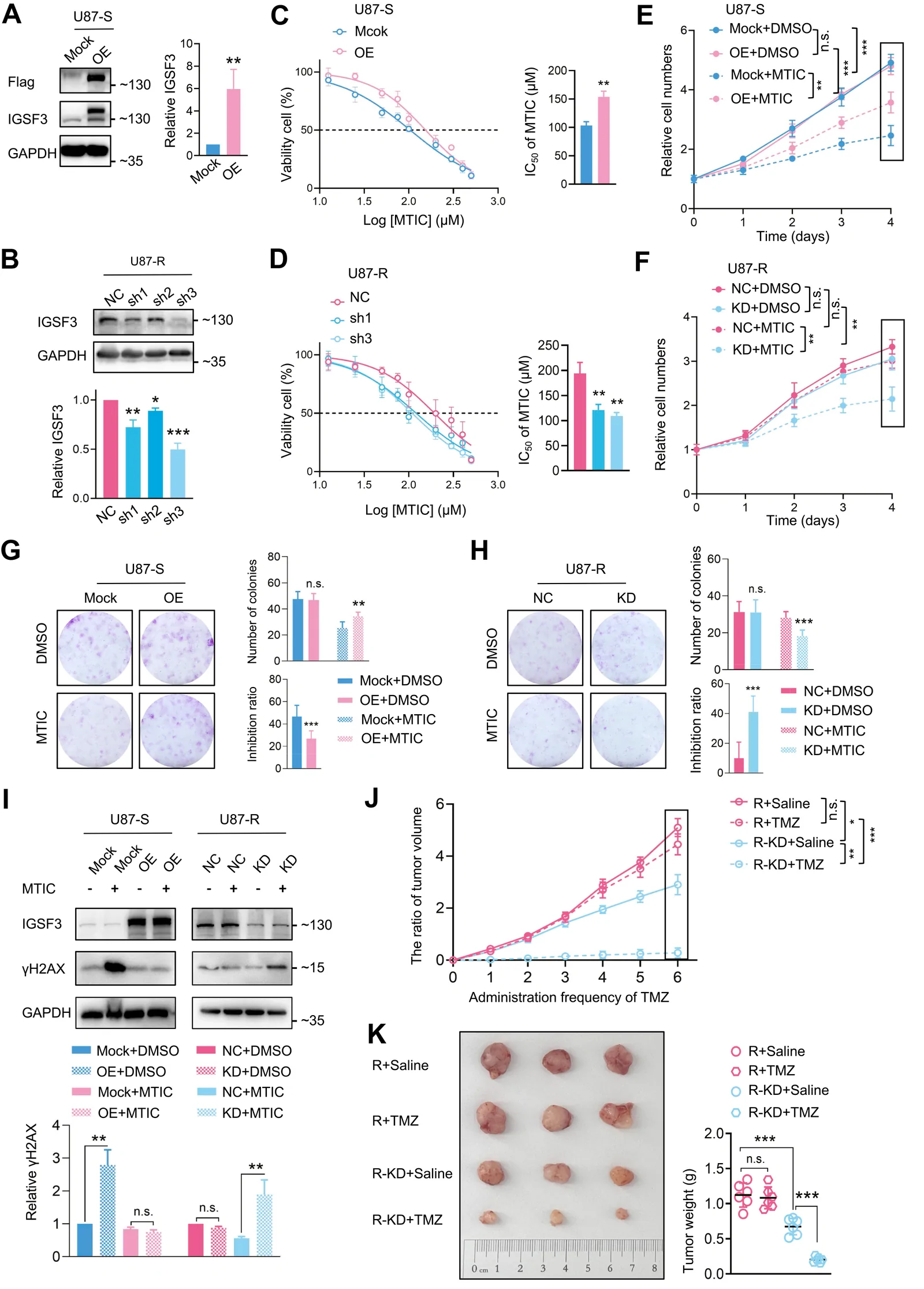
**IGSF3 facilitates resistance to MTIC and reduces DNA damage in GBM cells.***A* and *B*, Western blot validation of *IGSF3* overexpression in U87-S cells (*A*) and shRNA-mediated knockdown in U87-R cells (*B*). *C* and *D*, IC_50_ assay (*left*) and quantification (*right*) indicating different drug responses in U87-S Mock/*IGSF3*-OE cells (*C*) and U87-R NC/*IGSF3-*sh1/sh3 cells after 48 h of treatment with MTIC (*D*) (*n* = 3, ∗∗*p* < 0.01). *E* and *F*, CCK-8 viability curves of the indicated cells treated with DMSO or MTIC. *G* and *H*, representative images (*left*) and number of colony and inhibition ratio of colony formation assays. Cells were treated with DMSO or MTIC. *I*, Western blot analysis of γH2AX expression in U87-S (Mock/OE) and U87-R (NC/KD) cells following DMSO or MTIC treatment. *J*, the ratio of tumor volume of subcutaneous xenograft mouse models. The tumor volume ratio was calculated as (*Vt* − *V*_*0*_)/*V*_*0*_, where *V*_*0*_ represents the initial tumor volume at the start of treatment and Vt represents the tumor volume at the indicated time point. Data are presented as mean ± SD (*n* = 6, n.s., not significant, ∗*p* < 0.05, ∗∗*p* < 0.01). *K*, representative images (*left*) and tumor weight (*right*) of subcutaneous xenograft tumors harvested from nude mice (*n* = 6, n.s., not significant, ∗∗∗*p* < 0.001). DMSO, dimethyl sulfoxide; GBM, glioblastoma; IGSF, immunoglobulin superfamily; MTIC, 5-(3-methyltriazen-1-yl)-imidazole-4-carboxamide.

After establishing that IGSF3 is upregulated in resistant and recurrent GBM and preferentially expressed in GBM cells, we next tested whether IGSF3 functionally contributes to TMZ resistance. Therefore, we established stable *IGSF3*-overexpressing U87-S cells (Mock *versus* OE) and shRNA-mediated *IGSF3*-knockdown U87-R cells (NC *versus* KD). Success of the modifications was confirmed *via* Western blot (Fig. 4, *A* and *B*). Among the tested sequences, *IGSF3*-sh3 exhibited the highest silencing efficiency and was selected for subsequent experiments. Given that 5-(3-methyltriazen-1-yl)-imidazole-4-carboxamide (MTIC) is the active metabolite of TMZ, MTIC was used for *in vitro* drug sensitivity assays. IC_50_ analysis demonstrated that *IGSF3* overexpression significantly increased the resistance of U87-S cells to MTIC (Fig. 4*C*). Conversely, *IGSF3* depletion markedly sensitized U87-R cells to MTIC, with *IGSF3*-sh3-transfected cells exhibiting the most pronounced reduction in IC_50_ (Fig. 4*D*).

In CCK-8 viability assays, while *IGSF3* overexpression slightly slowed the baseline proliferation of U87-S cells, these cells maintained a significant survival advantage under MTIC treatment compared to Mock controls (Fig. 4*E*). In U87-R cells, MTIC alone had minimal impact on the NC group, but its inhibitory effect was prominently restored upon *IGSF3* knockdown (Fig. 4*F*). Similar results were observed in colony formation assays. Under vehicle conditions, clonogenic capacity did not differ significantly between Mock and OE groups, whereas *IGSF3*-OE cells formed significantly more colonies than Mock cells in the presence of MTIC (Fig. 4*G*). In resistant U87-R cells, *IGSF3* knockdown substantially increased the inhibitory rate of MTIC in resistant cells (Fig. 4*H*).

Because TMZ/MTIC cytotoxicity is mediated by DNA damage, we next assessed γH2AX accumulation as a marker of DNA double-strand breaks. MTIC-induced γH2AX levels were markedly attenuated in *IGSF3*-overexpressing U87-S cells, whereas *IGSF3* knockdown in U87-R cells led to a pronounced increase in γH2AX following MTIC exposure (Fig. 4*I*). These findings indicate that IGSF3 expression influences the magnitude of DNA damage accumulation under chemotherapeutic stress.

Finally, we validated these observations *in vivo* using a subcutaneous xenograft model. While TMZ treatment exerted limited inhibitory effects on U87-R-derived tumors, *IGSF3* knockdown significantly suppressed tumor growth. Importantly, combined IGSF3 depletion and TMZ treatment resulted in the greatest reductions in tumor volume and weight (Fig. 4, *J* and *K*). Collectively, these data demonstrate that IGSF3 enhances resistance to TMZ/MTIC both *in vitro* and *in vivo*, and that modulation of IGSF3 alters the extent of chemotherapy-induced DNA damage in GBM cells.

### Transcriptomic profiling identifies IGSF3-regulated metabolic pathways and downstream effectors

**Figure 5:**
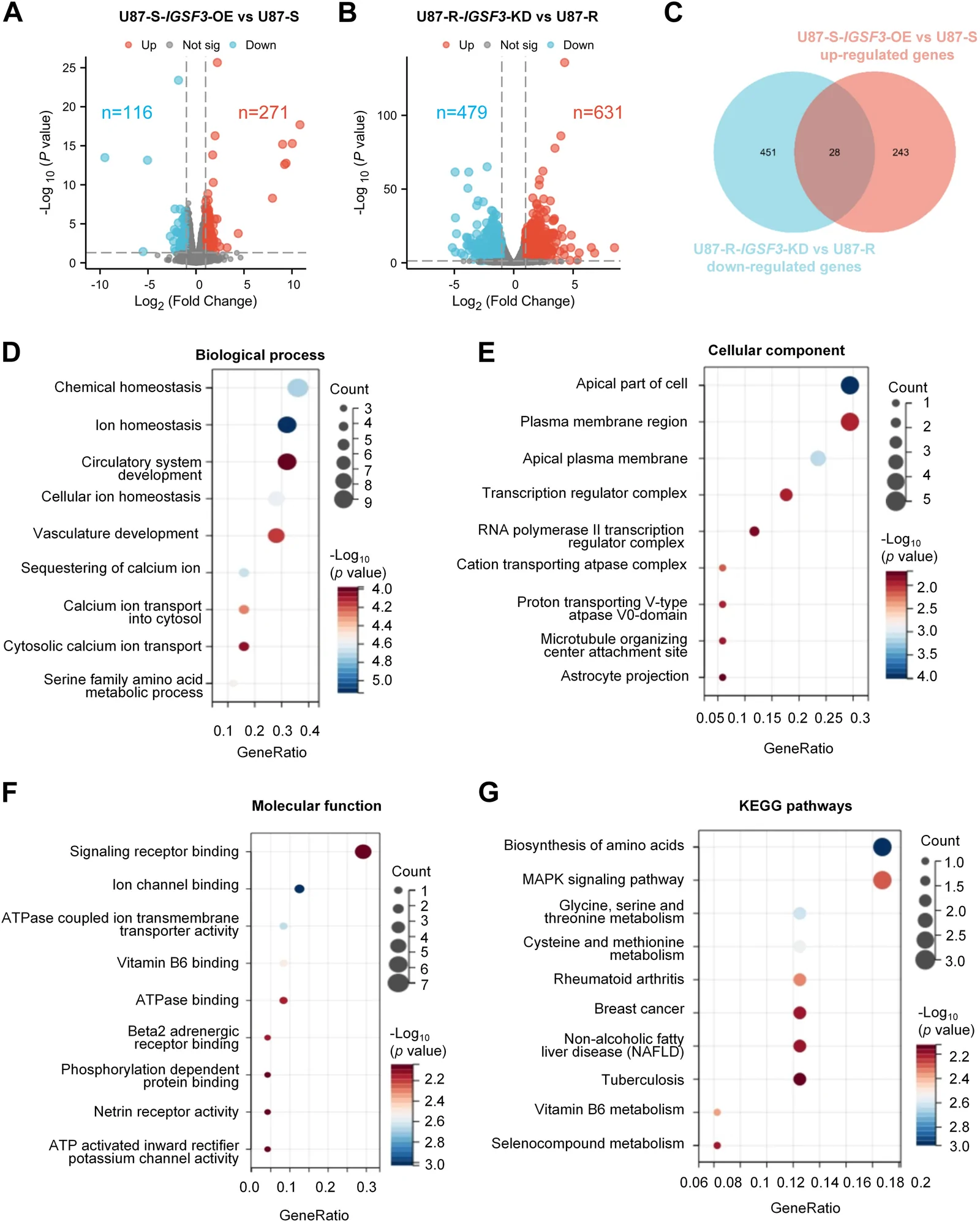
**Transcriptomic profiling identifies amino acid metabolism as a downstream target of *IGSF3*.***A* and *B*, volcano plots illustrating differentially expressed genes (DEGs) in U87-S *IGSF3*-OE *versus* Mock cells (*A*) and U87-R *IGSF3*-KD *versus* NC cells (*B*). Numbers of significantly upregulated (*red*) and downregulated (*blue*) genes are indicated. *C*, Venn diagram showing the 28 overlapping DEGs between the U87-S *IGSF3*-OE *versus* U87-S and U87-R *IGSF3*-KD *versus* U87-R groups. *D*, *E*, and *F*, GO enrichment analysis of common DEGs across Biological Process (*D*), Cellular Component (*E*), and Molecular Function (*F*) categories. *G*, KEGG pathway analysis highlighting the most significantly enriched pathways, with the biosynthesis of amino acids being the *top*-ranked term. IGSF, immunoglobulin superfamily.

To elucidate the downstream molecular mechanisms by which *IGSF3* contributes to TMZ resistance, we performed RNA-seq in *IGSF3*-overexpressing U87-S cells and *IGSF3*-knockdown U87-R cells. Differential expression analysis revealed extensive transcriptomic reprogramming in response to altered IGSF3 expression (|log_2_FC| ≥ 1 and *p* < 0.05). Specifically, 271 genes were upregulated and 116 downregulated in *IGSF3*-OE cells, whereas 631 genes were upregulated and 479 downregulated in *IGSF3*-depleted cells (Fig. 5, *A* and *B*). To identify genes consistently regulated by IGSF3, we intersected DEGs from both datasets and obtained a core set of 28 genes showing concordant expression changes with *IGSF3* modulation (Fig. 5*C*). These genes likely represent key downstream effectors mediating the functional impact of IGSF3 in TMZ resistance.

Functional enrichment analyses were then performed to characterize the biological significance of these IGSF3-responsive genes. GO analysis indicated enrichment in processes related to chemical homeostasis, ion transport, and receptor-binding functions (Fig. 5, *D*–*F*). Notably, KEGG pathway analysis revealed a pronounced enrichment in metabolic pathways, particularly amino acid biosynthesis and glycine, serine, and threonine metabolism (Fig. 5*G*).

Given the established role of amino acid metabolism in redox balance and cellular stress adaptation, these results suggest that IGSF3 may promote TMZ resistance by reprogramming metabolic homeostasis and activating amino acid-dependent adaptive pathways in GBM cells.

### IGSF3 regulates ASNS expression as a key downstream effector in acquired TMZ resistance

**Figure 6:**
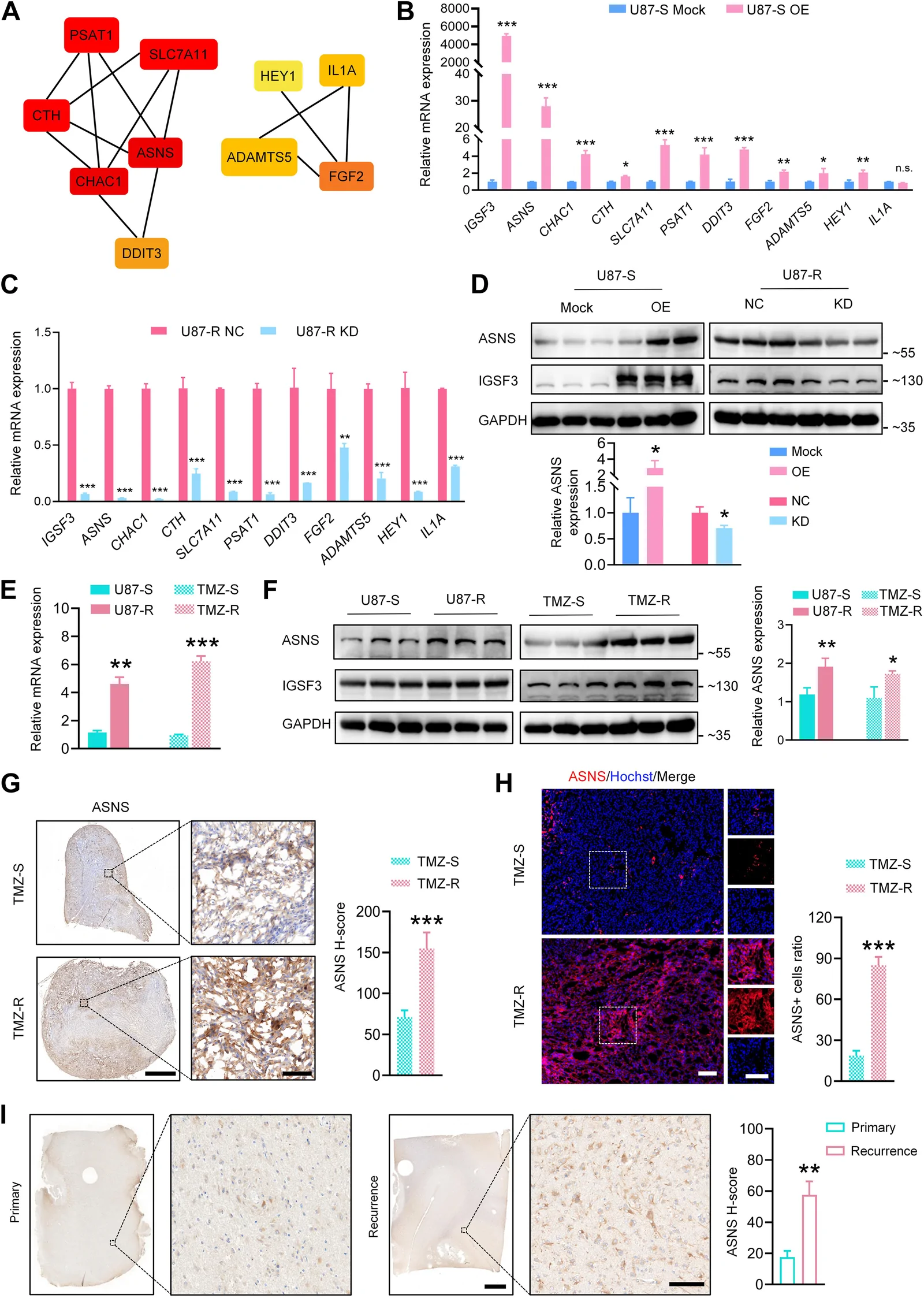
**Identification and validation of ASNS as a downstream target of IGSF3 in TMZ-resistant GBM.***A*, PPI network analysis of IGSF3-regulated common DEGs using the STRING database and Cytoscape. *B*, qRT-PCR validation of candidate genes in *IGSF3*-OE U87-S cells (*n* = 3, ∗*p* < 0.05, ∗∗*p* < 0.01, ∗∗∗*p* < 0.001). *C*, qRT-PCR validation of candidate genes in *IGSF3*-KD U87-R cells (*n* = 3, ∗∗*p* < 0.01, ∗∗∗*p* < 0.001). *D*, Western blot analysis of ASNS protein levels in *IGSF3*-OE U87-S and *IGSF3*-KD U87-R cells (*n* = 3, ∗*p* < 0.05). *E*, qRT-PCR analysis of *ASNS* expression in U87-S/R cells and TMZ-S/R tumor tissues (*n* = 3, ∗∗*p* < 0.01, ∗∗∗*p* < 0.001). *F*, Western blot analysis (*left*) and quantification (*right*) of ASNS protein levels in TMZ-S and TMZ-R cells and tumor tissues. *G* and *H*, representative IHC (*G*) and IF (*H*) images showing ASNS expression in TMZ-S and TMZ-R xenograft tissues (∗∗∗*p* < 0.001, the scale bars represent IHC: *left*-2 mm, *right*-100 μm; IF: 200 μm). *I*, IHC analysis of ASNS expression in paired clinical primary and recurrent GBM specimens with representative images and H-score quantification (the scale bars represent 2 mm (*left*) and 100 μm (*right*), ∗∗*p* < 0.01). ASNS, asparagine synthetase; DEG, differentially expressed gene; GBM, glioblastoma; IGSF, immunoglobulin superfamily; IHC, immunohistochemistry; qRT-PCR, quantitative reverse transcription PCR; TMZ-R, TMZ-resistant; TMZ-S, TMZ-sensitive.

To identify critical downstream mediators of IGSF3 signaling, we constructed a protein–protein interaction (PPI) network using the common DEGs derived from our RNA-seq analyses. Network analysis based on the STRING database and Cytoscape revealed a prominent hub cluster enriched for genes involved in amino acid metabolism, with *ASNS* emerging as a central node (Fig. 6*A*). To validate these observations, we performed qRT-PCR analysis of 10 candidate genes within the PPI network. Consistent with the RNA-seq data, all examined genes displayed expression changes concordant with *IGSF3* modulation, showing increased expression in *IGSF3*-overexpressing U87-S cells and decreased expression in *IGSF3*-knockdown U87-R cells (Fig. 6, *B* and *C*). Western blot analysis further confirmed that ASNS protein levels were markedly elevated upon *IGSF3* overexpression and substantially reduced following *IGSF3* knockdown (Fig. 6*D*), indicating that ASNS expression is positively regulated by IGSF3.

To further determine whether IGSF3-associated ASNS upregulation was accompanied by amino acid metabolic changes, we performed targeted measurement of selected amino acids in *IGSF3* overexpressing U87-S cells and *IGSF3* knockdown U87-R cells. The measured metabolites included amino acids directly related to the ASNS enzymatic reaction, including Asn, Asp, Gln and Glu, as well as representative amino acids involved in the enriched serine, glycine and threonine pathway and glutathione-associated oxidative stress buffering, including Ser, Gly, Thr and Cys. Among these amino acids, Asn and Glu showed the most consistent IGSF3-dependent pattern, increasing upon *IGSF3* overexpression in U87-S cells and decreasing after *IGSF3* knockdown in U87-R cells (Fig. S1, *A*–*H*). These data indicate that IGSF3-mediated ASNS upregulation is associated with altered amino acid metabolic output and further support the prioritization of the ASNS-Asn axis in acquired TMZ resistance.

We next examined ASNS expression in parental TMZ-S and TMZ-R models. *ASNS* mRNA expression was significantly increased in U87-R cells and TMZ-R tumor tissues compared with their sensitive counterparts (Fig. 6*E*), and this elevation was confirmed at the protein level by Western blotting (Fig. 6*F*). IHC and IF analyses of xenograft tissues further demonstrated strong ASNS staining in TMZ-R tumors relative to TMZ-S controls (Fig. 6, *G* and *H*). Importantly, analysis of clinical GBM specimens revealed significantly higher ASNS expression in recurrent tumors than in primary lesions (Fig. 6*I*). Together with the enrichment of amino acid biosynthesis pathways identified in our transcriptomic analyses, these findings identify ASNS as a key downstream target of IGSF3 and suggest that activation of the IGSF3-ASNS axis may contribute to metabolic adaptation and acquired TMZ resistance in GBM.

### ASNS-mediated asparagine biosynthesis is required for IGSF3-induced TMZ resistance

**Figure 7:**
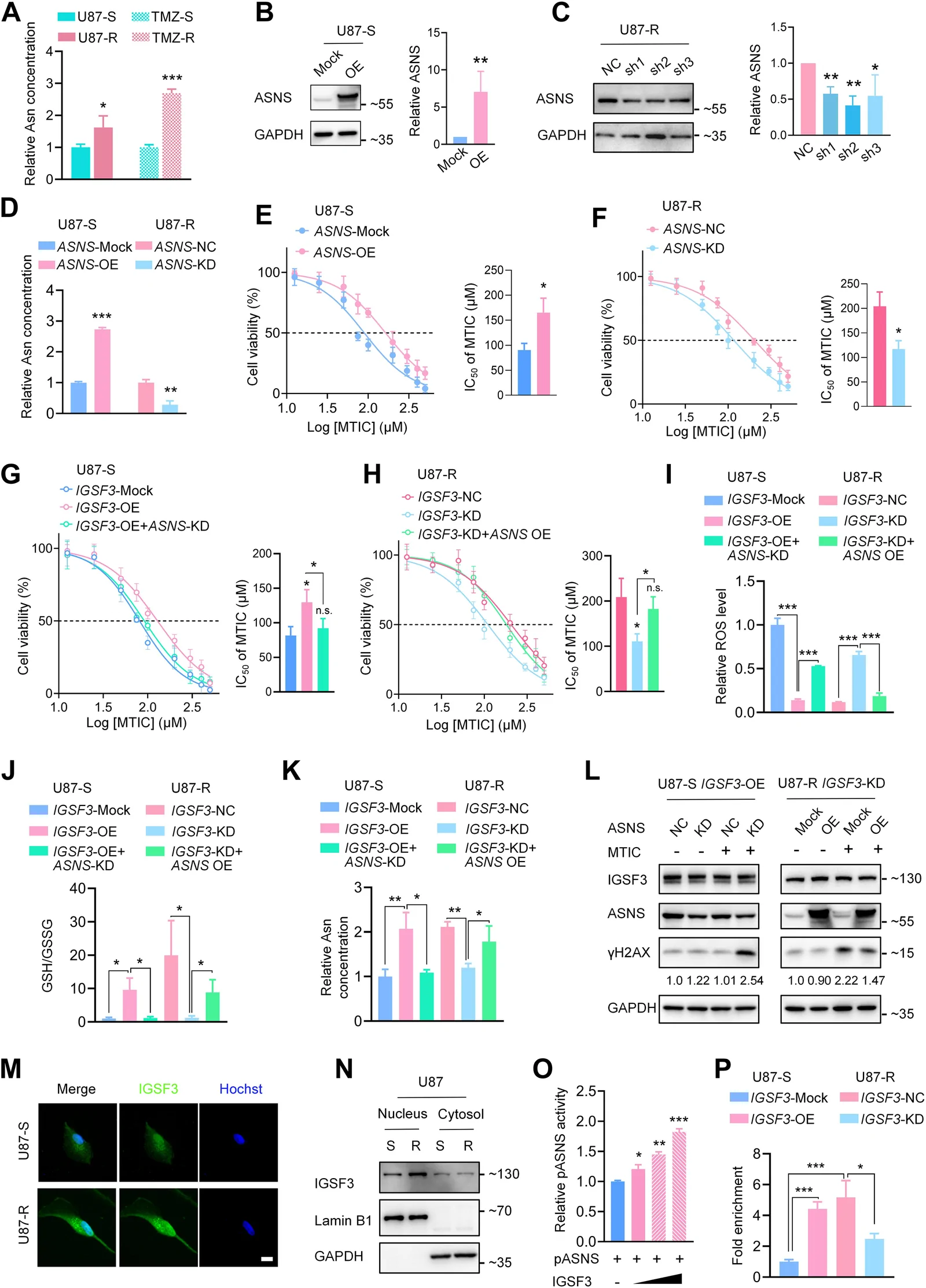
**IGSF3 promotes ASNS-dependent metabolic and oxidative stress adaptation to drive MTIC resistance.***A*, relative Asn concentration in U87-S/R cells and TMZ-S/R tumor tissues (*n* = 3, ∗∗*p* < 0.01, ∗∗∗*p* < 0.001). *B* and *C*, Western blot validation of *ASNS* overexpression in U87-S cells (*B*) and shRNA-mediated knockdown in U87-R cells (*C*) (*n* = 3, ∗*p* < 0.05, ∗∗*p* < 0.01, ∗∗∗*p* < 0.001). *D*, relative Asn concentration in the indicated *ASNS*-modified cell lines (*n* = 3, ∗∗*p* < 0.01, ∗∗∗*p* < 0.001). *E* and *F*, IC_50_ curves (*left*) and quantification (*right*) of MTIC in *ASNS*-overexpressing U87-S cells (*E*) and *ASNS*-knockdown U87-R cells (*F*) (*n* = 3, ∗*p* < 0.05). *G*, IC_50_ analysis (*left*) and quantification (*right*) of *IGSF3*-OE U87-S cells following transfection with control or *ASNS* shRNA (*n* = 3, ∗*p* < 0.05). *H*, IC_50_ analysis (*left*) and quantification (*right*) of *IGSF3*-KD U87-R cells following transfection with control or *ASNS* overexpression plasmid (*n* = 3, ∗*p* < 0.05). *I*, *J*, and *K*, relative intracellular ROS levels (*I*), GSH/GSSG ratio (*J*), and relative intracellular Asn concentration (*K*) in the indicated rescue models under MTIC treatment (*n* = 3, ∗*p* < 0.05, ∗∗*p* < 0.01, ∗∗∗*p* < 0.001). *L*, Western blot analysis of γH2AX expression in IGSF3-overexpressing U87-S cells with or without ASNS knockdown and in IGSF3-knockdown U87-R cells with or without *ASNS* overexpression under MTIC treatment. *M*, representative IF images showing the subcellular distribution of IGSF3 in U87-S and U87-R cells (the scale bar represents 20 μm). *N*, nuclear-cytoplasmic fractionation followed by Western blotting showing IGSF3 expression in cytoplasmic and nuclear fractions of U87-S and U87-R cells. *O*, ASNS promoter luciferase reporter assay in 293T cells cotransfected with increasing amounts of IGSF3 expression plasmid (*n* = 3, ∗*p* < 0.05, ∗∗*p* < 0.01, ∗∗∗*p* < 0.001). *P*, ChIP-qPCR analysis showing IGSF3 enrichment at the *ASNS* promoter in the indicated U87-S and U87-R cells (*n* = 3, ∗*p* < 0.05, ∗∗∗*p* < 0.001). ASNS, asparagine synthetase; IGSF, immunoglobulin superfamily; MTIC, 5-(3-methyltriazen-1-yl)-imidazole-4-carboxamide; ROS, reactive oxygen species; TMZ-R, TMZ-resistant; TMZ-S, TMZ-sensitive.

Having identified ASNS as an IGSF3-regulated metabolic effector, we next asked whether ASNS-dependent metabolic adaptation was functionally required for IGSF3-driven resistance. First, we quantified intracellular Asn levels in our experimental models. Asn content was significantly elevated in TMZ-R cells and tumor tissues compared with their sensitive counterparts (Fig. 7*A*), indicating enhanced asparagine biosynthesis during resistance acquisition. To assess the functional role of ASNS, we generated *ASNS*-overexpressing U87-S cells and *ASNS*-knockdown U87-R cells, with successful modulation confirmed by Western blot (Fig. 7, *B* and *C*). Consistent with its enzymatic activity, *ASNS* overexpression markedly increased intracellular Asn levels in U87-S cells, whereas *ASN*S depletion significantly reduced Asn levels in U87-R cells (Fig. 7*D*). We next evaluated whether ASNS directly influences chemotherapeutic sensitivity. IC_50_ analyses demonstrated that *ASNS* overexpression significantly increased resistance to MTIC in U87-S cells (Fig. 7*E*). In contrast, *ASNS* knockdown markedly sensitized U87-R cells to MTIC treatment (Fig. 7*F*), indicating that ASNS expression contributes functionally to the resistant phenotype.

To determine whether ASNS mediates the pro-resistance effect of IGSF3, rescue experiments were performed. Transfection of *ASNS* shRNA in *IGSF3*-overexpressing U87-S cells effectively restored sensitivity to MTIC (Fig. 7*G*). Conversely, overexpression of *ASNS* in *IGSF3*-depleted U87-R cells significantly reinstated MTIC resistance (Fig. 7*H*). We next investigated which ASNS-dependent cellular changes might connect IGSF3 expression to MTIC resistance. Given that ASNS regulates Asn biosynthesis and is linked to metabolic and oxidative stress adaptation in GBM (26), we examined reactive oxygen species (ROS) accumulation and the glutathione redox balance under MTIC treatment. In IGSF3-overexpressing U87-S cells, ASNS knockdown led to increased MTIC-induced ROS accumulation and a decreased GSH/GSSG ratio. Conversely, *ASNS* overexpression in *IGSF3* knockdown U87-R cells reduced ROS accumulation and partially restored the GSH/GSSG ratio under MTIC treatment (Figs. 7, *I*, *J* and S2, *A*, *B*). Concurrently, measurements of intracellular Asn confirmed that *ASNS* knockdown decreased, while *ASNS* overexpression restored, Asn levels in the respective rescue models (Fig. 7*K*). These results indicate that ASNS connects IGSF3 to both asparagine biosynthesis and oxidative stress buffering during MTIC treatment. To further determine whether ASNS mediates the effect of IGSF3 on MTIC-induced DNA damage, we examined γH2AX accumulation under MTIC treatment in these rescue settings. In *IGSF3*-depleted cells, MTIC markedly increased γH2AX levels, whereas overexpression of *ASNS* substantially attenuated this induction. Conversely, in *IGSF3*-overexpressing cells, MTIC induced only minimal γH2AX accumulation; however, silencing *ASNS* restored robust γH2AX induction following MTIC exposure (Fig. 7*L*). Together, these findings suggest that IGSF3 promotes MTIC resistance through ASNS-dependent Asn biosynthesis and oxidative stress adaptation, thereby reducing ROS accumulation and subsequent DNA damage signaling.

Having established that ASNS mediates the downstream pro-resistance effects of IGSF3, we next explored how IGSF3 regulates ASNS expression, given that IGSF3 has been mainly reported as a membrane-associated protein. IF staining revealed that IGSF3 was distributed not only on the membrane, but also in the cytoplasm and nucleus, with a stronger signal in U87-R cells than in U87-S cells (Fig. 7*M*). Consistently, subcellular fractionation confirmed the presence of IGSF3 in both nuclear and cytoplasmic fractions, and showed increased IGSF3 abundance in resistant cells (Fig. 7*N*). To determine whether IGSF3 affects ASNS transcriptional activity, we performed luciferase reporter assays in 293T cells by cotransfecting an *ASNS* promoter reporter with increasing amounts of IGSF3 expression plasmid. IGSF3 enhanced *ASNS* promoter activity in a dose-dependent manner (Fig. 7*O*), suggesting that IGSF3 positively regulates *ASNS* at the promoter level. Chromatin immunoprecipitation (ChIP)-qPCR further demonstrated enrichment of IGSF3 at the *ASNS* promoter. IGSF3 enrichment was increased in *IGSF3*-overexpressing U87-S cells and was reduced by *IGSF3* knockdown in U87-R cells (Fig. 7*P*), supporting a promoter-associated regulatory role of IGSF3 in ASNS transcription. Collectively, these data indicate that IGSF3 promotes ASNS-dependent asparagine biosynthesis and oxidative stress adaptation, at least in part through transcriptional regulation of ASNS.

### Tucatinib engages IGSF3 and suppresses the IGSF3-ASNS axis to enhance MTIC sensitivity in resistant GBM cells

**Figure 8:**
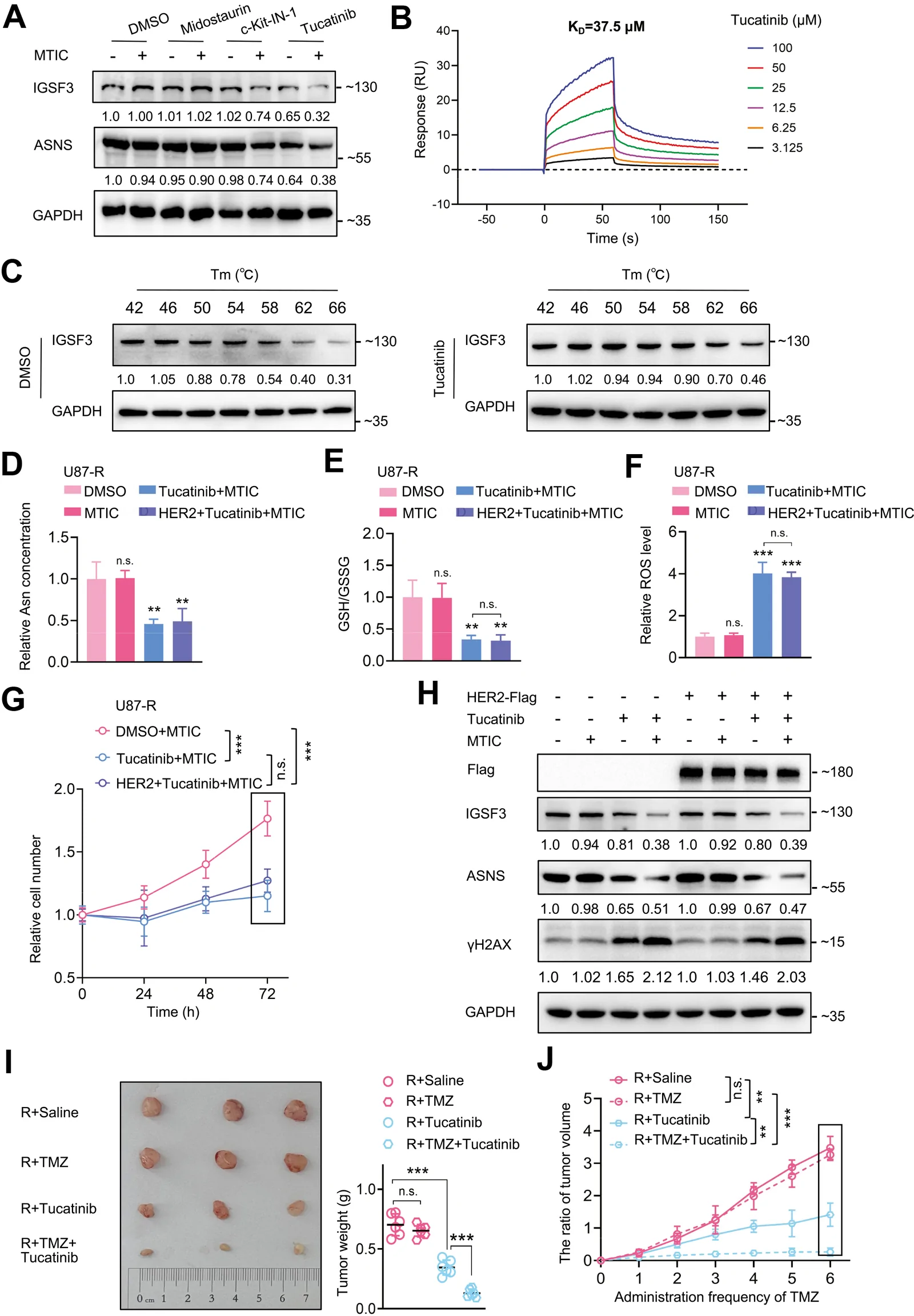
**Tucatinib engages IGSF3 and suppresses the IGSF3-ASNS axis to restore TMZ sensitivity in resistant GBM.***A*, Western blot analysis of IGSF3 and ASNS expression in U87-R cells treated with sub-IC_50_ concentrations of the three candidates. *B*, SPR analysis showing the binding of serially diluted Tucatinib to recombinant human IGSF3 protein. *C*, CETSA analysis showing the effect of Tucatinib on the thermal stability of cellular IGSF3. Relative band intensities are indicated below the corresponding lanes. *D*, *E*, and *F*, relative intracellular Asn levels (*D*), GSH/GSSG ratio (*E*), and relative intracellular ROS levels (*F*) in U87-R cells treated with DMSO, MTIC, Tucatinib plus MTIC, or HER2-OE plus Tucatinib and MTIC (n = 3, ∗∗*p* < 0.01, ∗∗∗*p* < 0.001). *G*, CCK-8 assay showing cell viability in the indicated treatment groups (∗∗*p* < 0.01). *H*, Western blot analysis of FLAG, IGSF3, ASNS, and γH2AX in U87-R cells with or without HER2-OE following treatment with Tucatinib or MTIC. *I* and *J*, representative images of subcutaneous xenograft tumors, tumor weights (*I*), and the ratio of tumor volume (*J*) from nude mice treated with saline, TMZ, Tucatinib, or the combination (*n* = 6, ∗∗*p* < 0.01, ∗∗∗*p* < 0.001). The tumor volume ratio was calculated as (*V*_*t*_ − *V*_*0*_)/*V*_*0*_, where *V*_*0*_ represents the initial tumor volume at the start of treatment and *V*_*t*_ represents the tumor volume at the indicated time point. Data are presented as mean ± SD. ASNS, asparagine synthetase; CETSA, cell thermal shift assay; DMSO, dimethyl sulfoxide; IGSF, immunoglobulin superfamily; MTIC, 5-(3-methyltriazen-1-yl)-imidazole-4-carboxamide; TMZ, temozolomide.

Because genetic disruption of the IGSF3-ASNS axis restored MTIC sensitivity, we next explored whether this pathway could be pharmacologically targeted. To explore the translational implications of targeting IGSF3, we performed *in silico* virtual screening of FDA-approved small molecules to identify compounds with potential binding affinity to IGSF3. The predicted three-dimensional structure of IGSF3, initial compound ranking, candidate binding models and IC_50_ assays are shown in Fig. S3, *A*–*D*. Among the top-ranked candidate compounds, Tucatinib produced the most evident suppression of IGSF3 and ASNS expression in TMZ-R U87-R cells, whereas c-Kit-IN-1 and Midostaurin showed comparatively weaker effects on this axis (Fig. 8*A*). Therefore, Tucatinib was selected for further mechanistic validation. We next examined whether Tucatinib directly engages IGSF3. surface plasmon resonance (SPR) analysis using recombinant human IGSF3 protein demonstrated concentration-dependent binding of Tucatinib to IGSF3 (Fig. 8*B*). Consistently, cell thermal shift assay (CETSA) showed that Tucatinib increased the thermal stability of cellular IGSF3, as reflected by increased retained IGSF3 signal at higher temperatures compared with the control condition (Fig. 8*C*). These results support physical target engagement between Tucatinib and IGSF3.

Because Tucatinib is known as a HER2-targeted tyrosine kinase inhibitor (29), we further evaluated whether its effects in our GBM models could be attributed solely to HER2. Basal HER2 expression was low in both U87-S and U87-R cells (Fig. S3*E*). We therefore introduced *HER2* overexpression into U87-R cells and examined the effects of Tucatinib under MTIC treatment. In U87-R cells, MTIC alone had limited effects on intracellular Asn levels, oxidative stress-related readouts and cell viability. In contrast, Tucatinib combined with MTIC reduced intracellular Asn levels, decreased the GSH/GSSG ratio, increased ROS accumulation and significantly suppressed cell viability (Figs. 8, *D*–*G* and S3, *F*, *G*). Importantly, *HER2* overexpression did not markedly reverse the effects of Tucatinib combine MTIC on Asn levels, GSH/GSSG ratio, ROS accumulation or cell viability (Fig. 8, *D*–*G*), suggesting that the effects of Tucatinib on the IGSF3-ASNS-associated metabolic and stress-related phenotype are not solely explained by HER2 expression. Moreover, Western blot analysis showed that Tucatinib reduced IGSF3 and ASNS expression and enhanced MTIC-induced γH2AX accumulation in U87-R cells. These effects were retained in HER2-overexpressing U87-R cells (Fig. 8*H*). The therapeutic potential of this combination strategy was further evaluated *in vivo*. In subcutaneous xenograft models derived from U87-R cells, TMZ monotherapy exerted limited antitumor effects, whereas Tucatinib treatment significantly suppressed tumor growth. Importantly, the combination of Tucatinib and TMZ resulted in the most pronounced reduction in tumor volume and weight (Fig. 8, *I* and *J*).

Collectively, these results identify Tucatinib as an IGSF3-engaging small-molecule candidate that suppresses the IGSF3-ASNS axis, disrupts oxidative stress adaptation, and enhances TMZ sensitivity in acquired TMZ resistant GBM models.

## Discussion

**Figure 9:**
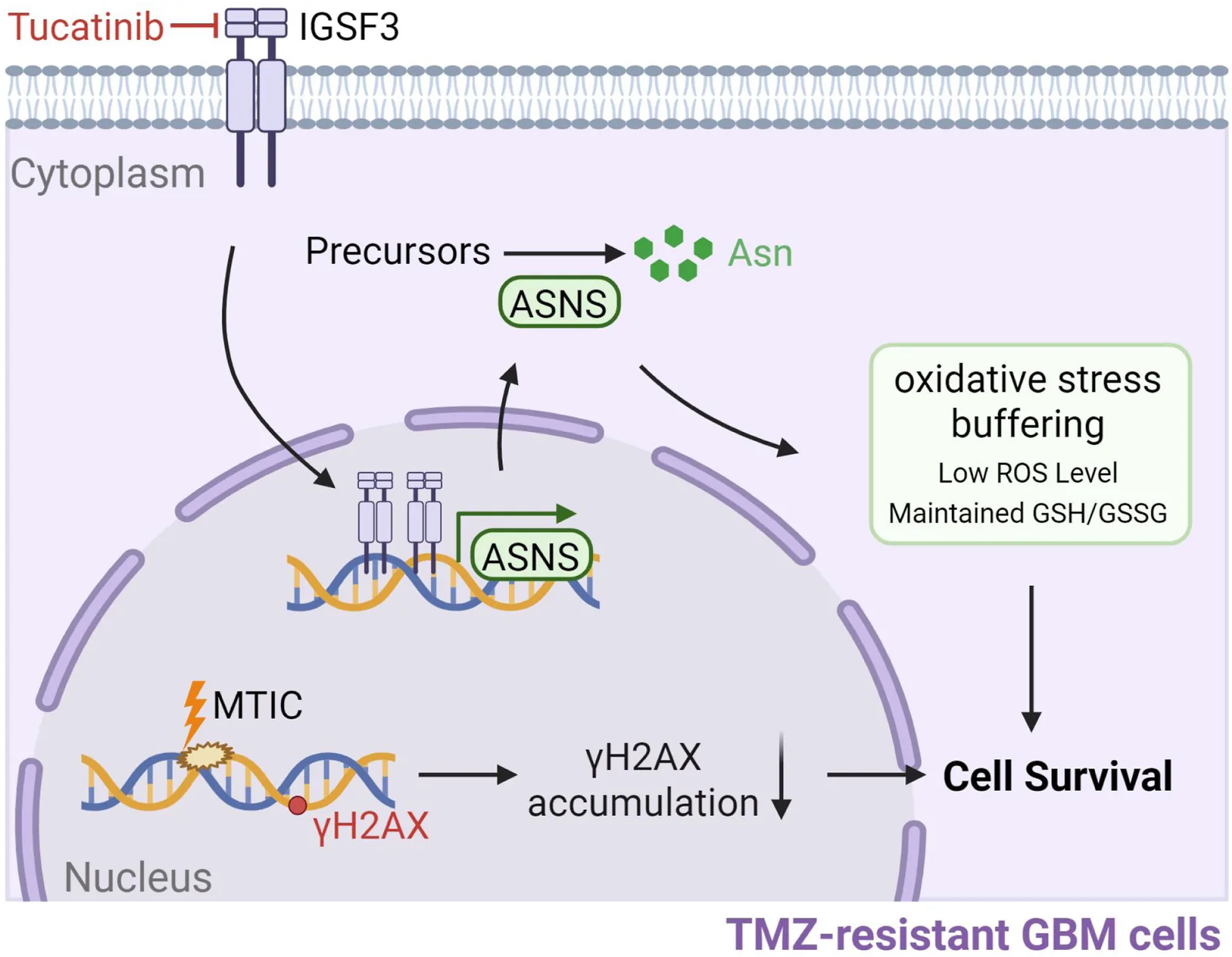
**Schematic model of the IGSF3-ASNS axis in acquired TMZ resistance in GBM.** In TMZ-resistant GBM, increased IGSF3 expression is associated with elevated ASNS expression and enhanced asparagine biosynthesis. This metabolic adaptation may reduce chemotherapy-induced oxidative stress and DNA damage accumulation, thereby promoting tumor cell survival under TMZ stress, thereby contributing to acquired TMZ resistance. Tucatinib is included as a candidate small-molecule modulator that may suppress the IGSF3-ASNS axis and enhance TMZ sensitivity. This panel was generated with BioRender.com. ASNS, asparagine synthetase; GBM, glioblastoma; IGSF, immunoglobulin superfamily; TMZ, temozolomide.

In the present study, we identified IGSF3 as a previously unrecognized mediator of acquired TMZ resistance in GBM. By integrating transcriptomic data from two independent acquired-resistance models, we prioritized IGSF3 as a robustly upregulated candidate associated with poor clinical outcome. We then demonstrated that IGSF3 was preferentially expressed in malignant glioma cells at single-nucleus resolution and became enriched in recurrent GBM, where a higher proportion of IGSF3-positive tumor cells correlated with worse survival. Functional experiments further showed that IGSF3 enhanced resistance to MTIC/TMZ *in vitro* and *in vivo* while reducing chemotherapy-induced γH2AX accumulation. Transcriptomic profiling revealed a prominent metabolic program downstream of IGSF3, leading to the identification of ASNS as a central effector. Mechanistically, IGSF3 was detected in the nuclear compartment and promoted ASNS expression through promoter-associated regulation. This IGSF3-ASNS axis increased Asn production, reduced MTIC-induced ROS accumulation, maintained the GSH/GSSG ratio and limited γH2AX accumulation, thereby helping GBM cells adapt to TMZ-related therapeutic stress. ASNS rescue experiments further demonstrated that ASNS was functionally required for the pro-resistance effect of IGSF3. Finally, Tucatinib engaged IGSF3, suppressed the IGSF3-ASNS axis, disrupted oxidative stress adaptation and enhanced TMZ sensitivity in resistant GBM models. Together, these findings establish a tumor-intrinsic metabolic mechanism underlying acquired chemoresistance in GBM and provide a potentially actionable therapeutic axis (Fig. 9).

Although IGSF3 has not been extensively studied in cancer, accumulating evidence suggests that it functions as a pro-tumorigenic molecule and, in certain tumor contexts, may also contribute to immunosuppression (14, 30, 31). In glioma, the most prominent prior study linked IGSF3 to tumor progression through potassium dysregulation and neuron–glioma interactions, showing that IGSF3 can promote neuronal hyperexcitability and glioma growth (15, 16). Our findings are broadly consistent with these reports in that they also position IGSF3 as an adverse and functionally important molecule in GBM. Our findings extend the current understanding of IGSF3. Rather than being solely associated with glioma progression, IGSF3 is functionally linked to acquired TMZ resistance. Its expression is largely restricted to malignant glioma cells, supporting a tumor-intrinsic role, and its enrichment in recurrent lesions suggests positive selection under therapeutic pressure. Together, these observations identify IGSF3 as a clinically relevant driver of chemoresistance in GBM.

A central mechanistic finding of this study is the identification of ASNS as a critical downstream effector of IGSF3, and that IGSF3 may promote ASNS expression through a nuclear-associated, promoter-related regulator mechanism. ASNS catalyzes the ATP-dependent conversion of aspartate and glutamine into Asn and has increasingly been recognized as an important node in nutrient stress adaptation and tumor metabolic plasticity (32, 33, 34). In GBM, previous studies have shown that ASNS is frequently amplified or highly expressed and is associated with poor prognosis, increased metabolic flexibility, maintenance of redox homeostasis, and resistance to cellular stress, including radiation-related injury (26). Additional work has suggested that disrupting Asn metabolism may impair the growth of both TMZ-S and TMZ-R GBM cells, supporting the functional importance of this pathway in treatment adaptation (35, 36). Our study builds directly on this body of literature by demonstrating that the IGSF3-ASNS axis is functionally required for acquired TMZ resistance. Specifically, we found that ASNS occupied a central position in the IGSF3-regulated protein interaction network, was consistently upregulated in resistant cells, resistant tumors, and recurrent patient samples, and closely mirrored the expression pattern of IGSF3. Targeted amino acid measurements further showed that IGSF3 modulation was accompanied by changes in selected amino acids related to ASNS-associated metabolism, with Asn showing the most consistent IGSF3-dependent pattern. An interesting aspect of our findings is that IGSF3, although generally regarded as a membrane-associated IGSF protein, was also detectable in the nuclear fraction of U87 cells. Together with the promoter luciferase and ChIP-qPCR results, this provides a potential mechanistic explanation for how IGSF3 positively regulates ASNS expression. Although our data support a promoter-associated regulatory role of IGSF3, they do not establish IGSF3 as a canonical transcription factor or prove that it directly binds DNA. Whether IGSF3 is recruited to the ASNS promoter through nuclear cofactors, and how it translocates to or is retained in the nuclear compartment, remain important questions for future studies. Functionally, ASNS was required for the pro-resistance effect of IGSF3. Gain- and loss-of-function experiments showed that ASNS altered MTIC sensitivity, while rescue experiments demonstrated that *ASN*S knockdown reversed the resistant phenotype induced by *IGSF3* overexpression and that ectopic *ASNS* overexpression partially restored resistance in IGSF3-depleted cells. Importantly, ASNS-dependent effects were not limited to drug-sensitivity readouts. The IGSF3-ASNS axis increased Asn production, reduced MTIC-induced ROS accumulation, maintained the GSH/GSSG ratio and limited γH2AX accumulation. Thus, IGSF3-ASNS-dependent metabolic adaptation may help GBM cells tolerate MTIC-induced oxidative and genotoxic stress, thereby reducing downstream DNA damage signaling and promoting therapeutic resistance. This is consistent with previous evidence showing that increasing DNA damage burden can restore chemosensitivity in resistant tumor cells (37, 38, 39, 40, 41), further supporting the notion that modulation of treatment-induced genotoxic stress is a critical determinant of therapeutic response. Notably, other IGSF3-responsive genes identified in our transcriptomic analyses, including PSAT1 (42), CTH (43), SLC7A11 (44), CHAC1 (45), and DDIT3 (46), are also linked to amino acid metabolism and stress adaptation, implying that IGSF3 may coordinate a broader metabolic survival program centered on ASNS.

Virtual screening has been widely applied in anticancer drug discovery as an efficient strategy for prioritizing candidate compounds and accelerating the identification of targetable small molecules (47, 48). Our analyses further suggest that this pathway may be pharmacologically tractable. Tucatinib is a clinically approved, HER2-selective tyrosine kinase inhibitor with established systemic activity and clinically meaningful central nervous system efficacy in HER2-positive malignancies, particularly in the setting of brain metastases (29, 49, 50, 51). In our study, virtual screening nominated Tucatinib as a candidate compound with predicted binding to IGSF3. SPR and CETSA assays further provided experimental support for this predicted interaction, indicating that Tucatinib can engage IGSF3. Functionally, Tucatinib suppressed the IGSF3-ASNS axis, reduced Asn production, disrupted glutathione redox balance, increased ROS accumulation, and restored MTIC/TMZ sensitivity in resistant GBM cells. Importantly, although HER2 was overexpressed as a control condition, Tucatinib retained its ability to suppress the IGSF3-ASNS axis and enhance chemotherapy sensitivity.

This study still has some limitations. First, although our findings were supported by clinical specimens, and public single-nucleus data and *in vivo* experiments, the mechanistic work was primarily conducted in U87-based acquired-resistance models; validation in additional patient-derived GBM cells, organoids, orthotopic PDX and recurrent GBM-derived models will be important to establish broader generalizability. Second, our clinical analysis was primarily based on overall survival, because progression-free survival or disease-free survival information was incomplete in the analyzed public cohort. Therefore, the relationship between IGSF3 expression and GBM progression or recurrence requires further validation in cohorts with matched longitudinal follow-up data. Third, although our data support nuclear localization and promoter-associated regulation of ASNS by IGSF3, the precise mechanism by which IGSF3 enters the nucleus and regulates promoter activity remains unresolved. Fourth, our amino acid measurements were targeted analyses of selected metabolites rather than comprehensive metabolomics or isotope-tracing experiments. Thus, the broader metabolic flux regulated by IGSF3-ASNS remains to be defined. Finally, although Tucatinib treatment reduced IGSF3 and ASNS expression and attenuated the IGSF3-ASNS-associated resistant phenotype, the precise mechanism by which Tucatinib suppresses this axis remains unclear. In addition, although HER2 overexpression did not abolish the effects of Tucatinib in our U87-R model, further validation in additional GBM models with different HER2 expression backgrounds will be required to fully clarify the contribution of HER2 to Tucatinib-mediated suppression of the IGSF3-ASNS axis. Despite these limitations, our study identifies IGSF3 as a tumor-intrinsic driver of acquired TMZ resistance and establishes ASNS-dependent asparagine biosynthesis and oxidative stress adaptation as a critical metabolic mechanism underlying this phenotype.

## Experimental procedures

### Online sources and processing

Datasets GSE151680 and GSE174554 were obtained from the Gene Expression Omnibus database. Our in-house RNA-seq dataset was generated from our previously established TMZ-S and TMZ-R U87-derived GBM models and has been described in our previous study (27). GSE151680 was used as a bulk transcriptomic dataset comparing parental U87 cells with acquired TMZ-R U87 cells (U87TR), whereas GSE174554 was used as a single-nucleus RNA-seq dataset of paired primary and recurrent IDH-WT GBM specimens. For GSE151680, the expression matrix was processed using R software (version 4.5.1). Probe IDs were converted to official gene symbols according to the corresponding platform annotation file. When multiple probes mapped to the same gene, the average expression value was used. Expression values were log_2_-transformed and normalized before differential expression analysis. DEGs between U87 and U87TR cells were identified using the limma package, with the cutoff set as |log_2_FC| > 1.5 and *p* < 0.05. This threshold was selected to identify genes with statistically significant and biologically meaningful expression changes for subsequent candidate screening and experimental validation. For GSE174554, only samples containing complete barcodes, features and matrix files were included. Raw count matrices were imported into Seurat, and Seurat objects were generated with min.cells = 3 and min.features = 200. Low-quality cells/nuclei were removed according to the distributions of nFeature_RNA, nCount_RNA, percent.mt and percent.ribo. Specifically, cells/nuclei with fewer than 200 detected genes, mitochondrial gene percentage greater than 2.5%, or more than 7000 detected genes were excluded. The expression matrix was normalized using the LogNormalize method with a scale factor of 10,000. The top 3000 highly variable genes were identified using the vst method, and MALAT1, NEAT1 and ribosomal genes were removed from the highly variable gene set to reduce the influence of highly abundant nonspecific transcripts. Data were scaled with nCount_RNA and percent.ribo regressed out. PCA was then performed, and the first 20 principal components were used for downstream analysis. Harmony was applied to correct sample-level batch effects using sample identity as the batch variable. The first 20 Harmony dimensions were used for neighbor graph construction, clustering and UMAP/t-SNE visualization. Clustering was tested at multiple resolutions, including 0.1, 0.2, 0.3, 0.5, 0.8 and 1.0. A resolution of 0.3 was selected because it preserved the major cell populations without excessive oversegmentation. Marker genes for each cluster were identified using FindAllMarkers with only.pos = TRUE, min.pct = 0.25 and logFC.threshold = 0.25; genes with adjusted *p* < 0.05 were considered significant markers. Cell-type annotation was determined by integrating cluster marker genes, literature-reported GBM single-cell markers (28, 52, 53). SingleR annotation was performed based on the Human Primary Cell Atlas. Volcano plots were generated using the Xiantao Academic online platform (https://www.xiantao.love/products).

### Functional enrichment analysis

GO enrichment analysis was performed using Metascape (54), which integrates biological process, molecular function, and cellular component annotations. Enriched terms were considered significant if *p* < 0.05 and gene count > 3. KEGG pathway analysis was carried out using the Xiantao Academic online platform.

### Construction of PPI network

The PPI network was constructed utilizing the Search Tool for the Retrieval of Interacting Genes (STRING) database (55). A high-confidence interaction threshold was applied by setting the combined score to > 0.7. The resulting interaction network was imported into Cytoscape for visualization. Hub genes were identified using the cytoHubba plugin, and genes were ranked according to the degree algorithm.

### Analysis of gene clinical outcomes

GlioVis (56) was used to evaluate the prognostic significance of *SYT13*, *CHI3L1* and *IGSF3* in CGGA glioma cohorts. Patients were stratified into high- and low-expression groups according to the median expression level of each gene. Kaplan-Meier survival curves were performed to evaluate the association between gene expression and overall survival, and differences between groups were evaluated using the log-rank test.

### Cell culture and reagents

The U87-S and U87-R cells were obtained from TMZ-S and TMZ-R U87 mouse models as previously described (27). The human glioma cell lines U251 and LN229, the human hepatocellular carcinoma cells HepG2 and the human embryonic kidney cell line 293T, were obtained from the Cell Bank of Type Culture Collection of the Chinese Academy of Sciences. Cells were routinely tested for *mycoplasma* contamination and used within limited passages after thawing. All cells were cultured in high-glucose Dulbecco's modified Eagle's medium (DMEM) supplemented with 10% fetal bovine serum (Invitrogen,) and 1% penicillin–streptomycin (Hyclone) at 37 °C in a humidified incubator containing 5% CO_2_. MTIC (Cat. No. HY-119696, MedChemExpress), TMZ (Cat. No. S1237, Selleckchem), Tucatinib (Cat. No. T2364, TargetMol), Midostaurin (Cat. No. T3211, TargetMol), and c-Kit-IN-1 (Cat. No. T4332, TargetMol) were dissolved according to the manufacturers’ instructions and stored as aliquots to avoid repeated freeze-thaw cycles.

### Animal tumor models and treatment

All animal experiments were approved by the Institutional Animal Care and Use Committee of The Affiliated Wuxi People's Hospital of Nanjing Medical University (No. (2023)142). Six-week-old male BALB/c nude mice were purchased from Cavens and maintained under specific pathogen-free conditions. The *in vivo* mouse models were established as previously described (27). Briefly, GBM cells were suspended in sterile PBS or serum-free DMEM, and 5 × 10^6^ cells were injected subcutaneously into the right flank of each mouse. Tumor length and width were measured with calipers, and tumor volume was calculated using the formula: volume = length × width^2^/2. Body weight and tumor volume were monitored regularly. When tumors reached approximately 100 to 150 mm^3^, mice were randomly assigned to treatment groups. TMZ and Tucatinib were administered at 20 mg/kg and 30 mg/kg respectively *via* oral gavage every 48 h for 14 days, whereas control mice received vehicle. Importantly, the two drugs were administered on alternating days to avoid overlapping drug exposure, ensuring intermittent and staggered treatment throughout the experimental period. At the experimental endpoint, mice were euthanized, and tumors were collected for histological and molecular analyses. The number of mice in each group is indicated in the corresponding figure legends. All animal experiments were approved bythe medical ethics committee of The Affiliated Wuxi People's Hospital of Nanjing Medical University (No. (2023)142) and were complied with the Chinese national standard GB/T 35892-2018 (Guideline for the Ethical Review of Laboratory Animal Welfare).

### Plasmids and primers

Full-length cDNA encoding human IGSF3 (pLV3-CMV-IGSF3-FLAG-Puro), ASNS (pLV3-CMV-ASNS-FLAG-Puro), HER2 (pCDNA3.1-ERBB2-3×FLAG) and ASNS-promoter reporter plasmid (pASNS-Fluc-SV40-hRluc-Neo) were constructed and purchased from MiaoLingBio. The short hairpin RNA (shRNA) sequences of IGSF3 and ASNS were listed in Table S1, which were constructed and purchased from MiaoLingBio. The primers used for qPCR were listed in Table S1.

### qRT-PCR

The tumor cells and tissues total RNA was extracted with TRIzol reagent (Thermo Fisher Scientific). cDNA was synthesized using the Hiscript III Reverse Transcriptase (Vazyme). qRT-PCR was performed with ChamQ SYBR qPCR Master Mix (Vazyme) on a Real-Time System (Bioer). Data were analysis by the comparative Ct (2^−ΔΔCt^) method with amplification efficiency correction. The primer sequences used for amplification are listed in Table S1.

### Cell growth and colony formation assay

Corresponding cells were seeded at a density of 1 × 10^3^ cells per well in 96-well plates and cultured in complete DMEM. Cell viability was measured at the indicated time points using the Cell Counting Kit-8 (CCK-8; Vazyme). For the colony formation assay, 500 U87 cells per well were plated in six-well plates and maintained in complete DMEM. After 14 days of incubation, colonies were fixed with 4% paraformaldehyde and stained with 0.1% crystal violet (Sangon). Colony numbers were counted under an inverted microscope and analyzed using ImageJ software.

### Cell growth inhibition assay

Cytotoxicity was assessed by seeding Corresponding cells at a density of 3 × 10^3^ cells per well in 96-well plates. Cells were exposed to MTIC (active metabolite of TMZ), Tucatinib (T2364), Midostaurin (T3211) or c-Kit-IN-1 (T4332) was in complete DMEM for 48 h. Cell viability was evaluated using CCK-8. Dose-response relationships were modeled by logistic regression, and IC_50_ values were determined as the concentration of drug causing a 50% decrease in cell viability.

### Western blot

For Western blot detection, cells and tumor tissues were lysed in radioimmunoprecipitation assay buffer (Sigma-Aldrich) containing protease and phosphatase inhibitor cocktails (Pierce). Equal amounts of protein were separated on 10 to 12% SDS-PAGE gels (Vazyme) and transferred to nitrocellulose membranes using the Novex gel transfer system (Invitrogen). Membranes were blocked and then incubated overnight at 4 °C with primary antibodies, after which species-specific HRP-conjugated secondary antibodies (Proteintech) were applied for 1 h at RT. Target bands were visualized using Tanon 5200 Multi (Tanon). Detailed information on the primary antibodies used for Western blot analysis was provided in Table S2. The uncropped original images are also shown in Fig. S4.

### IHC and IF staining

IHC and IF staining were performed by Wuhan Servicebio Technology Co., Ltd, and staining intensity was quantified using the Aipathwell image analysis system to evaluate expression patterns across samples. Detailed information on the primary antibodies used for IHC and IF analysis was provided in Table S2. All clinical samples corresponding experiments were approved by the medical ethics committee of The Affiliated Wuxi People's Hospital of Nanjing Medical University (KY25180) and were performed in accordance with the Declaration of Helsinki.

### RNA-seq

RNA-seq and differential expression analysis. Total RNA was extracted from U87-S cells with or without IGSF3 overexpression and from U87-R cells with or without IGSF3 knockdown using TRIzol reagent (Takara, Cat. 9109) according to the manufacturer’s instructions. Qualified RNA samples were used for library construction and subjected to RNA-seq on the Illumina NovaSeq platform. Raw FASTQ reads were filtered to remove adapter sequences and low-quality reads to obtain clean reads. Gene-level read counts were generated using featureCounts, and gene expression levels were normalized and expressed as FPKM values. Differential expression analysis between groups was performed using the DESeq2 package. Genes with *p* < 0.05 and |log_2_FC| ≥ 1 were considered differentially expressed. RNA-seq and primary bioinformatic analyses were performed by Tsingke Biotechnology Co., Ltd.

### Intracellular amino acid content detection assay

Intracellular levels of selected amino acids, including Asn, Asp, Gln, glutamate (Glu), threonine (Thr), cysteine (Cys), serine (Ser) and glycine (Gly), were measured using commercially available assay kits (Shanghai Enzyme-linked Biotechnology Co., Ltd) according to the manufacturers’ instructions. Briefly, cells were collected, washed with cold PBS, processed using the corresponding kit-compatible extraction or sample preparation buffers, and centrifuged to remove insoluble debris. The resulting supernatants were used for amino acid quantification. Asn was measured using an enzymatic assay in which Asn was hydrolyzed by asparaginase to generate aspartate and NH4+, followed by glutamate dehydrogenase-mediated oxidation of NADH in the presence of α-ketoglutarate; Asn content was calculated based on the decrease in absorbance at 340 nm. Asp, Gln, Glu, Thr, Ser and Gly were detected using ELISA-based colorimetric assays, with absorbance measured at 450 nm. Cys was measured using a colorimetric microplate assay based on the reduction of phosphotungstic acid, with absorbance measured at 600 nm. Concentrations were calculated from standard curves and expressed as relative levels after normalization to the corresponding control group.

### Intracellular ROS detection assay

Intracellular ROS levels were measured using a DCFH-DA ROS detection kit (Cat. No. S0033S, Beyotime Biotechnology) according to the manufacturer’s standard protocols. Briefly, indicated cells with different treatments were collected and washed gently with precooled PBS, then incubated with diluted DCFH-DA working solution in a serum-free medium at 37 °C in the dark for 20 min. After incubation, residual unbound probes were thoroughly removed by PBS washing. Subsequently, the intracellular fluorescence intensity indicative of endogenous ROS levels was dynamically measured at sequential time points using Multiskan FC (Thermo fisher Scientific), with the excitation wavelength set at 488 nm and the emission wavelength at 525 nm. The relative fluorescence intensity of each group was calculated and normalized to the corresponding baseline control to evaluate dynamic alterations in intracellular oxidative stress before and after stimulation. All experiments were independently performed in triplicate to ensure stable and reproducible results.

### Intracellular GSH and GSSG detection assay

Intracellular GSH and GSSG levels were determined using a GSH and GSSG detection kit (Cat. No. S0053, Beyotime Biotechnology) according to the manufacturer’s instructions. Briefly, indicated cells with different treatments were harvested and rinsed with precooled PBS. Cell pellets were resuspended in the kit-supplied lysis buffer and fully lysed on ice. After high-speed centrifugation, the supernatants were collected for detection. Total glutathione (GSH + GSSG) and GSSG contents were detected in parallel, and the absorbance of each sample was measured using Multiskan (Thermo fisher Scientific) at 412 nm. Standard curves were applied to calculate the concentrations of total glutathione and GSSG, and the relative GSH level was further deduced. The intracellular GSH/GSSG ratio was statistically analyzed to reflect the dynamic redox balance and oxidative stress status. All experiments were performed independently in triplicate to ensure experimental reproducibility.

### Nuclear and cytoplasmic protein extraction

Cytoplasmic and nuclear protein fractions were isolated using the Nuclear and Cytoplasmic Extraction Kit (Cat. No. E101-01, Vazyme Biotech) in accordance with the manufacturer’s standard protocols. Briefly, treated cells were harvested and washed with precooled PBS. Cell pellets were resuspended in cytoplasmic lysis buffer supplemented with protease inhibitor cocktail, incubated on ice, and centrifuged to collect cytoplasmic protein supernatant. The remaining nuclear precipitates were further lysed with nuclear extraction buffer on ice, followed by high-speed centrifugation to obtain purified nuclear protein solution. Equal quantities of cytoplasmic and nuclear protein were subjected to Western blot analysis for IGSF3 detection. GAPDH served as the cytoplasmic internal reference, and Lamin B1 was used as the nuclear loading control. All experiments were performed in biological triplicate to ensure reproducibility.

### Virtual screening

The amino acid sequence of human IGSF3 was obtained from the UniProt database (https://www.uniprot.org/uniprotkb/O75054/entry), and its full-length three-dimensional structure was predicted using AlphaFold3. Virtual screening was performed by Phadcalc company.

### SPR analysis

The binding affinity between small molecule Tucatinib (T2364, TargetMol) and recombinant human IGSF3 domain (aa 20-1124, C-terminal 10 x His tag, MedChemExpress, HY-P76412) was measured using a Biacore T200 system. Recombinant human IGSF3 was immobilized onto a CM5 sensor chip *via* amine coupling. Serial dilutions of T2364 were injected at a flow rate of 30 μl/min. Equilibrium dissociation constant (K_D_) and kinetic constants were calculated using a 1:1 Langmuir binding model after subtracting the reference flow cell signal. Data were analyzed using Biacore T200 Evaluation Software 3.1.

### CETSA

CETSA was conducted to assess cellular target engagement between Tucatinib and endogenous IGSF3. Briefly, cells were incubated with equal concentrations of DMSO (vehicle control) or Tucatinib for sufficient binding saturation. Cells were then collected and resuspended in ice-cold PBS containing protease inhibitor cocktail. The cell suspension was aliquoted equally and subjected to a temperature gradient of 42 °C, 46 °C, 50 °C, 54 °C, 58 °C, 62 °C, and 66 °C for 3 min using a thermal cycler, followed by immediate incubation on ice for 5 min to stop thermal denaturation. All samples were lysed *via* repeated freeze-thaw cycles, and soluble protein supernatants were harvested after high-speed centrifugation. The remaining soluble IGSF3 protein levels in DMSO and Tucatinib groups at each temperature were determined by Western blotting. Relative IGSF3 protein abundance was quantified and normalized to the corresponding 42 °C control group.

### Dual-luciferase reporter assay

ASNS transcriptional activity was evaluated by a luciferase reporter system. Briefly, cells were transfected with the ASNS-promoter reporter plasmid (MiaoLingBio) and concentration gradient of full-length cDNA encoding human IGSF3. After 36 h of transfection, the culture medium was discarded, and cells were fully lysed using cell lysis buffer on ice and measured using the Dual Luciferase Reporter Gene Assay Kit (Beyotime Biotechnology).

### ChIP assay

ChIP assays were performed using the ChIP Assay Kit (Beyotime Biotechnology) according to the manufacturer’s instructions. Briefly, 2 × 10^7^ cells and 5 μg anti-IGSF3 antibody were used for each ChIP. The rabbit IgG was applied as negative control. After reversal of cross-links and DNA purification, the samples were used for quantitative real-time PCR with specific primers targeting gene promoters (Table S1, Supporting Information). ChIP efficiency was calculated as the percentage of input chromatin (% Input) and fold enrichment relative to the IgG control group. The relative binding enrichment of IGSF3 on ASNS gene promoter was quantified and normalized to the corresponding input samples to eliminate experimental variations. All ChIP experiments were conducted in biological triplicate to ensure the reliability and reproducibility of protein-DNA binding results.

### Statistical analysis

Data were expressed as mean ± SD. Comparisons between two groups were performed using an unpaired two-tailed Student’s *t* test. Comparisons among multiple groups were performed using ANOVA followed by Newman-Keuls’ multiple comparison test. Tumor volume ratio was calculated as (*V*_*t*_ − *V*_*0*_)/*V*_*0*_, statistical comparisons of tumor growth curves among four groups were performed using repeated-measures one-way ANOVA followed by Tukey’s *post hoc* multiple comparison test. Survival differences were evaluated using the Kaplan-Meier method and log-rank test. Associations between categorical variables were analyzed using the χ^2^ test, and correlations were assessed using Spearman’s rank correlation analysis. IC_50_ values were calculated by nonlinear regression analysis of dose-response curves and compared between groups as indicated. Differences were considered statistically significant at *p* < 0.05. Statistical analyses and graphing were performed using SPSS 16.0 (IBM) and GraphPad Prism 10.0 (GraphPad Software), respectively.

## Data availability

The publicly available datasets analyzed during this study (GSE151680 and GSE174554) are available in the NCBI Gene Expression Omnibus repository. The RNA-seq dataset was deposited in the Sequence Read Archive repository at NCBI under the accession number PRJNA1478155. The datasets used and analyzed during the current study are available from the corresponding author upon reasonable request.

## Supporting information

This article contains supporting information.

## Conflict of interest

The authors declare that they have no conflicts of interest with the contents of this article.
