# Supplementary material for: IGSF3 drives acquired temozolomide resistance in glioblastoma through ASNS-dependent asparagine biosynthesis
Source: J Biol Chem. 2026 Jul 22;302(9):113359. doi: 10.1016/j.jbc.2026.113359 (PMC13499201; doi:10.1016/j.jbc.2026.113359)
Supplement: Supplementary file 1 [file mmc1.pdf]

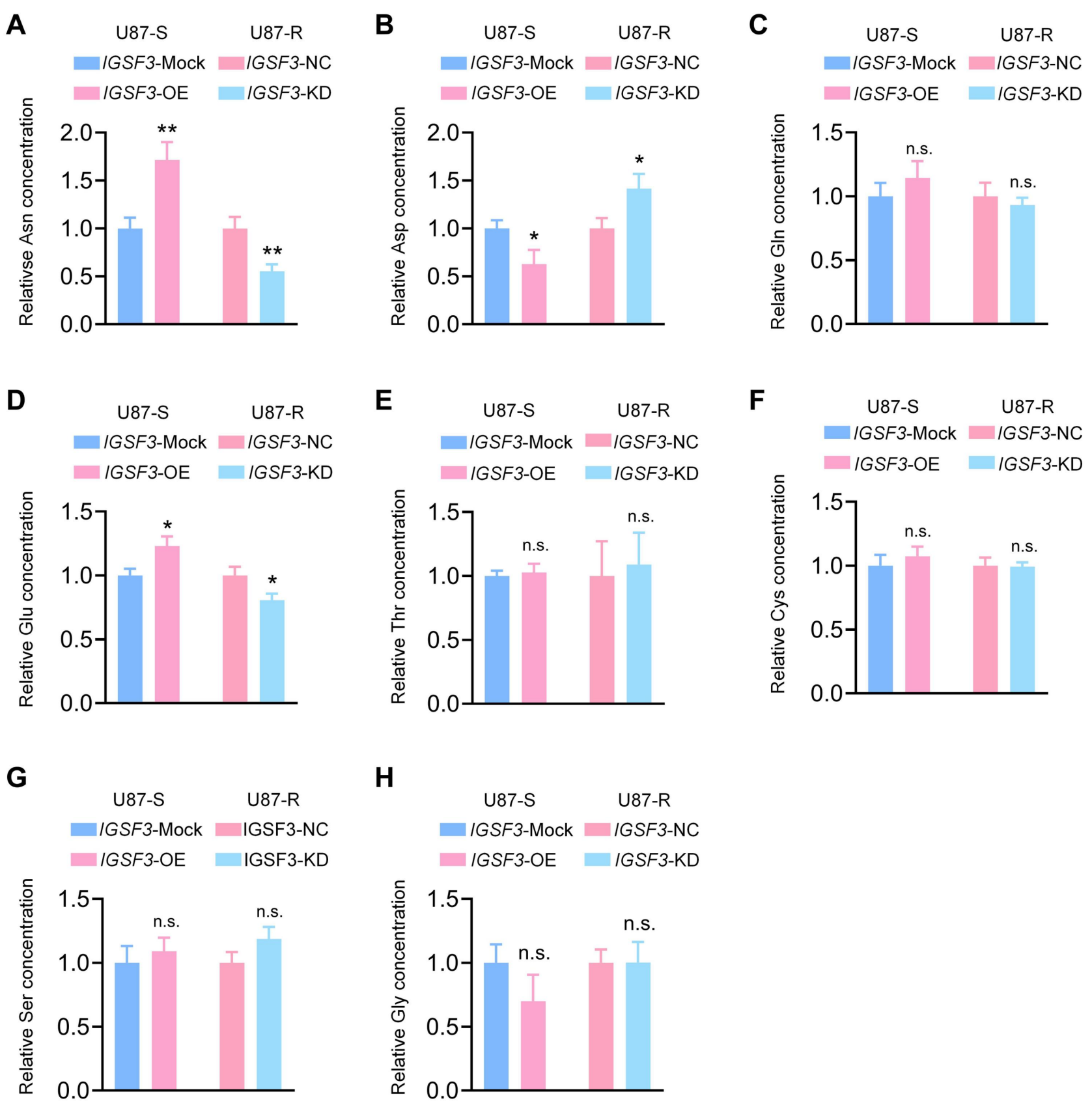

Supplementary Figure 1. Targeted measurement of selected amino acids in *IGSF3*-modified U87-S and U87-R cells.

A-H, Relative intracellular levels of selected amino acids in U87-S *IGSF3*-Mock/OE cells and U87-R *IGSF3*-NC/KD cells. The measured amino acids included Asn (**A**), Asp (**B**), Gln (**C**), Glu (**D**), Thr (**E**), Cys (**F**), Ser (**G**), and Gly (**H**) (n = 3, n.s., no significance, *$p < 0.05$, **$p < 0.01$).

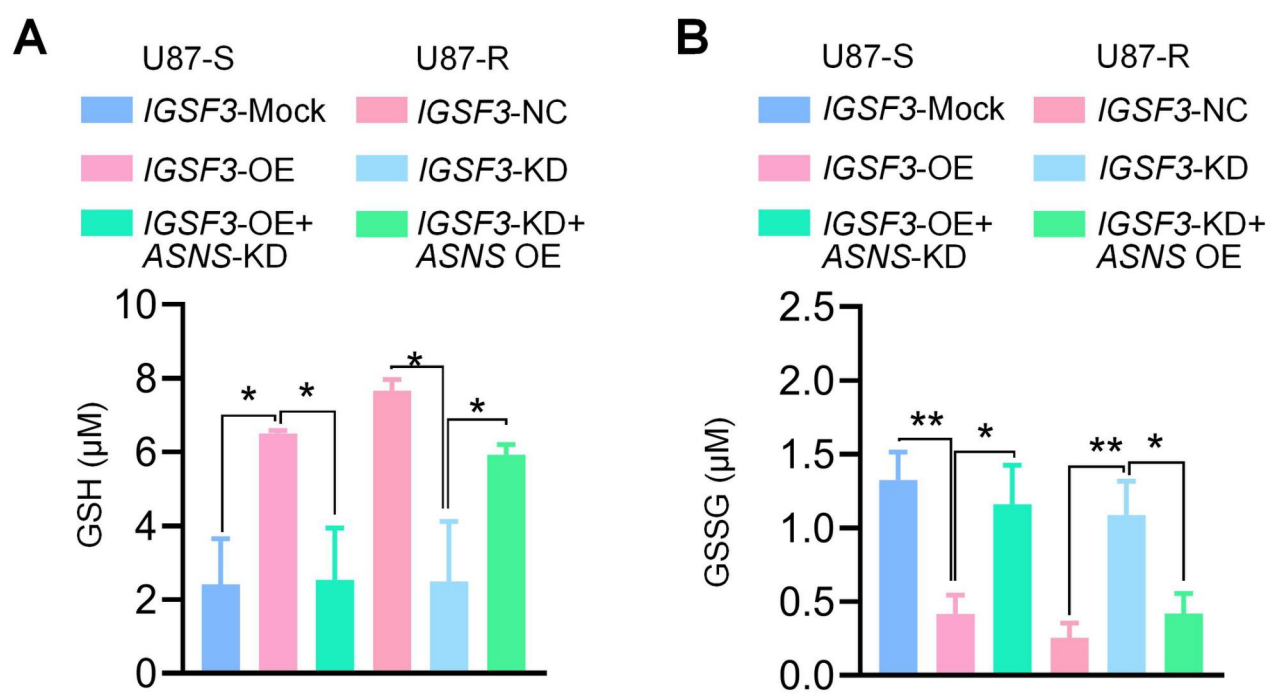

Supplementary Figure 2. GSH and GSSG levels in *ASNS* rescue models under MTIC treatment.

A-B, GSH (**A**) and GSSG (**B**) concentration in the indicated rescue models under MTIC treatment (n = 3, *$p < 0.05$, **$p < 0.01$). These measurements correspond to the GSH/GSSG ratio analysis shown in Fig. 7J.

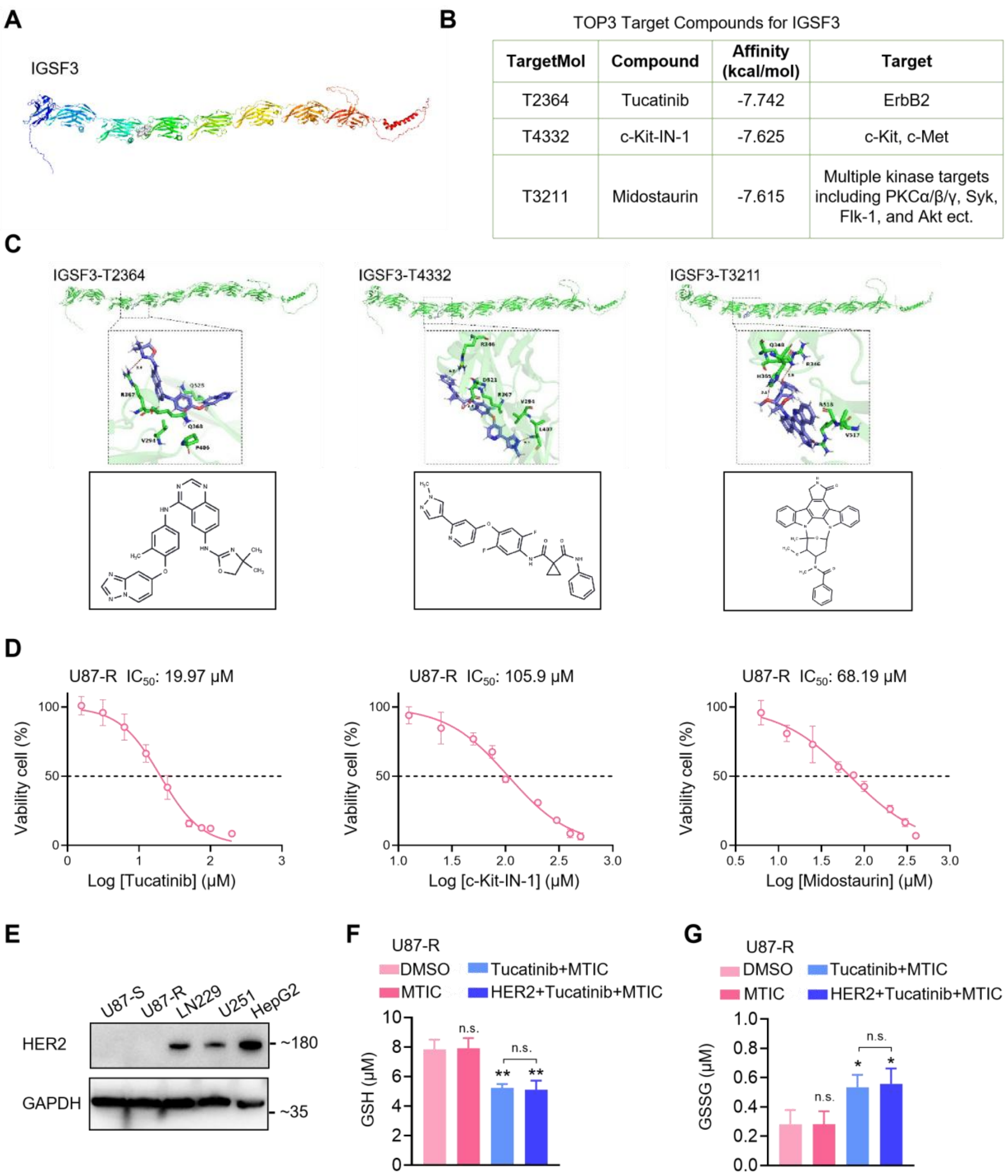

TargetMol	Compound	Affinity (kcal/mol)	Target
T2364	Tucatinib	-7.742	ErbB2
T4332	c-Kit-IN-1	-7.625	c-Kit, c-Met
T3211	Midostaurin	-7.615	Multiple kinase targets including PKCα/β/γ, Syk, Flk-1, and Akt ect.

Supplementary Figure 3. Virtual screening of candidate IGSF3-targeting compounds and supporting validation of Tucatinib activity.

A, Predicted 3D structure of the IGSF3 protein obtained from the AlphaFold database. **B**, Top three candidate drugs, including Tucatinib, c-Kit-IN-1, and Midostaurin, identified through virtual screening based on predicted binding affinity scores. **C**, Chemical structures and 3D molecular docking models showing the predicted binding conformations of the candidate drugs within the IGSF3 protein pocket. **D**, IC_{50} curves showing the inhibitory effects of the three candidate drugs on U87-R cells after 48 h of treatment. **E**, Western blot analysis of HER2 expression in U87-S, U87-R, LN229, U251, and HepG2 cells. **F-G**, GSH (**F**) and GSSG (**G**) concentration in U87-R cells treated with DMSO, MTIC, Tucatinib plus MTIC, or HER2 overexpression plus Tucatinib and MTIC (n = 3, n.s., no significance, *$p < 0.05$, **$p < 0.01$).

Figure 2F

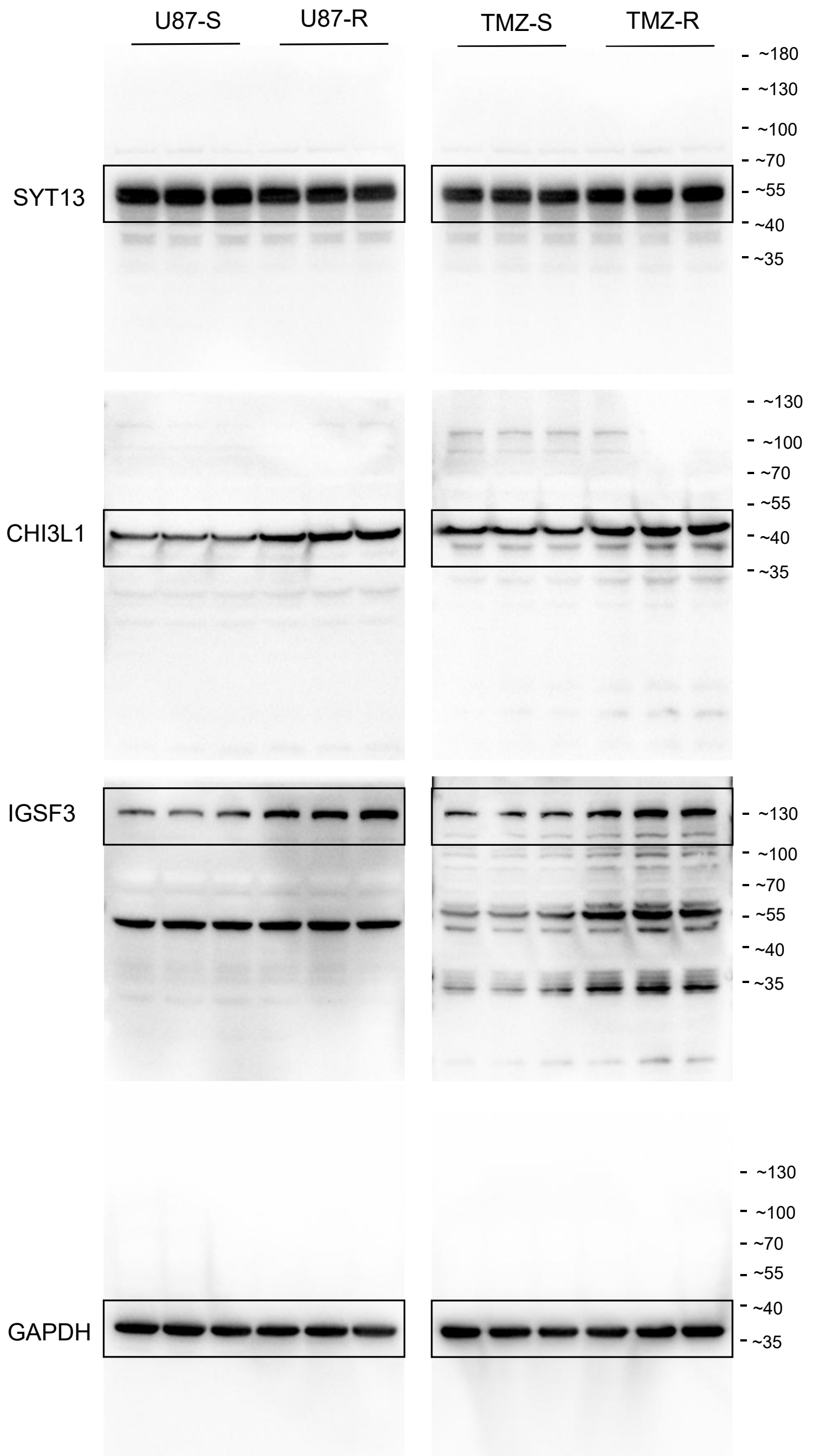

Figure 4A

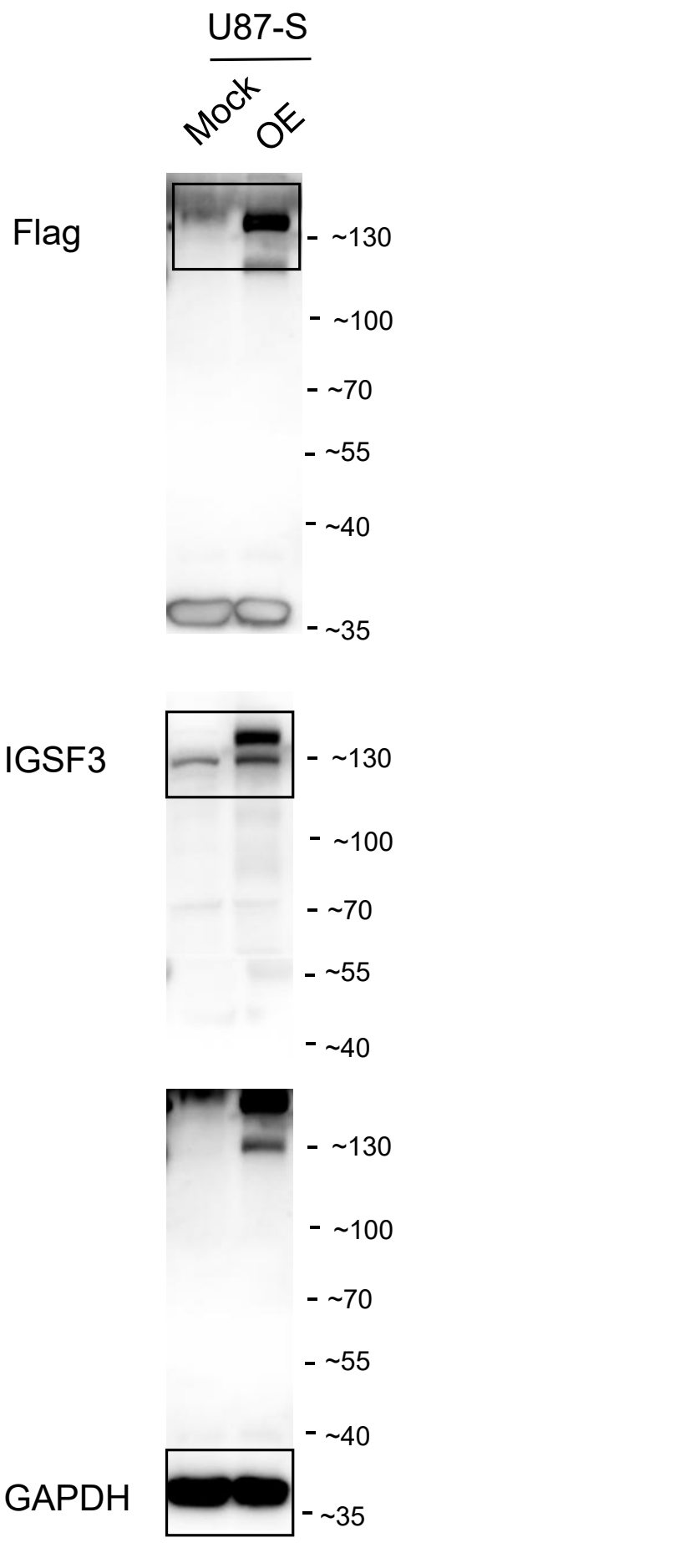

Figure 4B

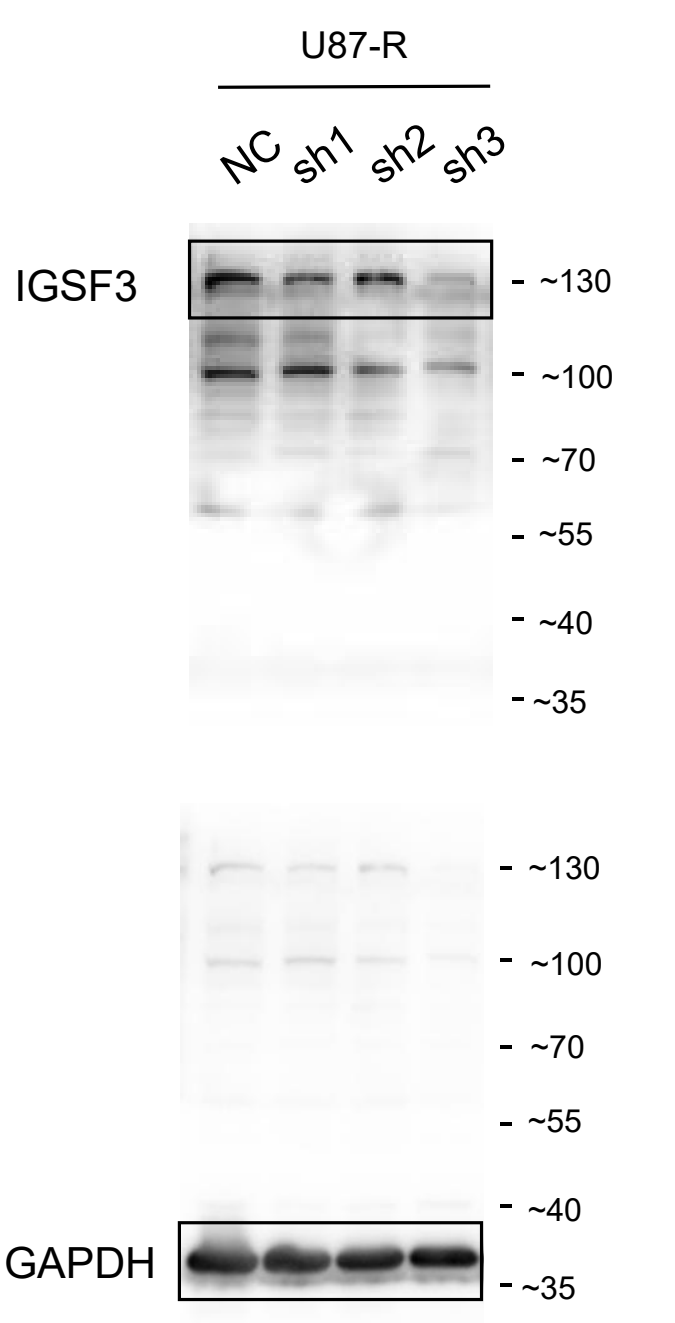

Supplementary Figure 4

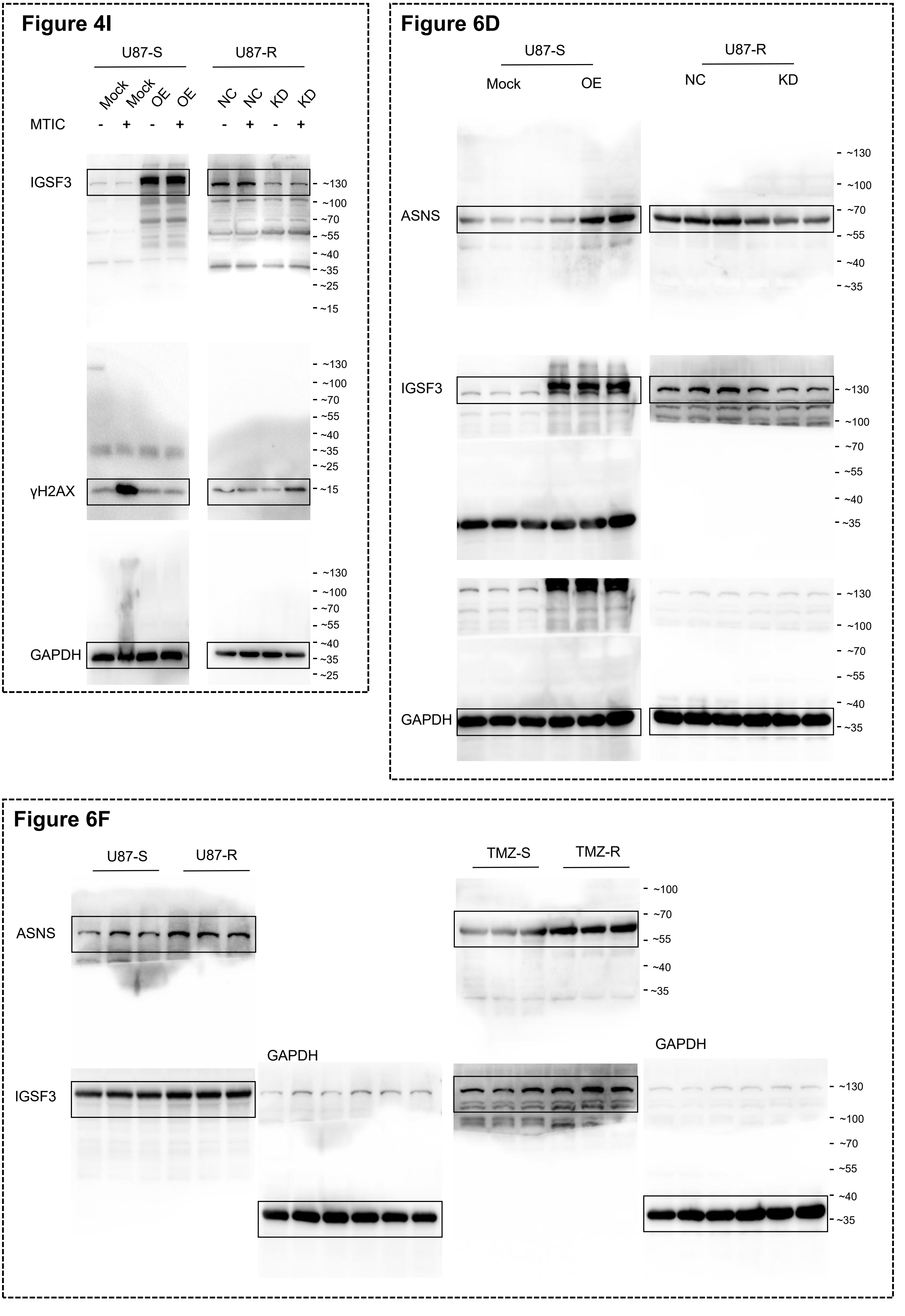

Supplementary Figure 4

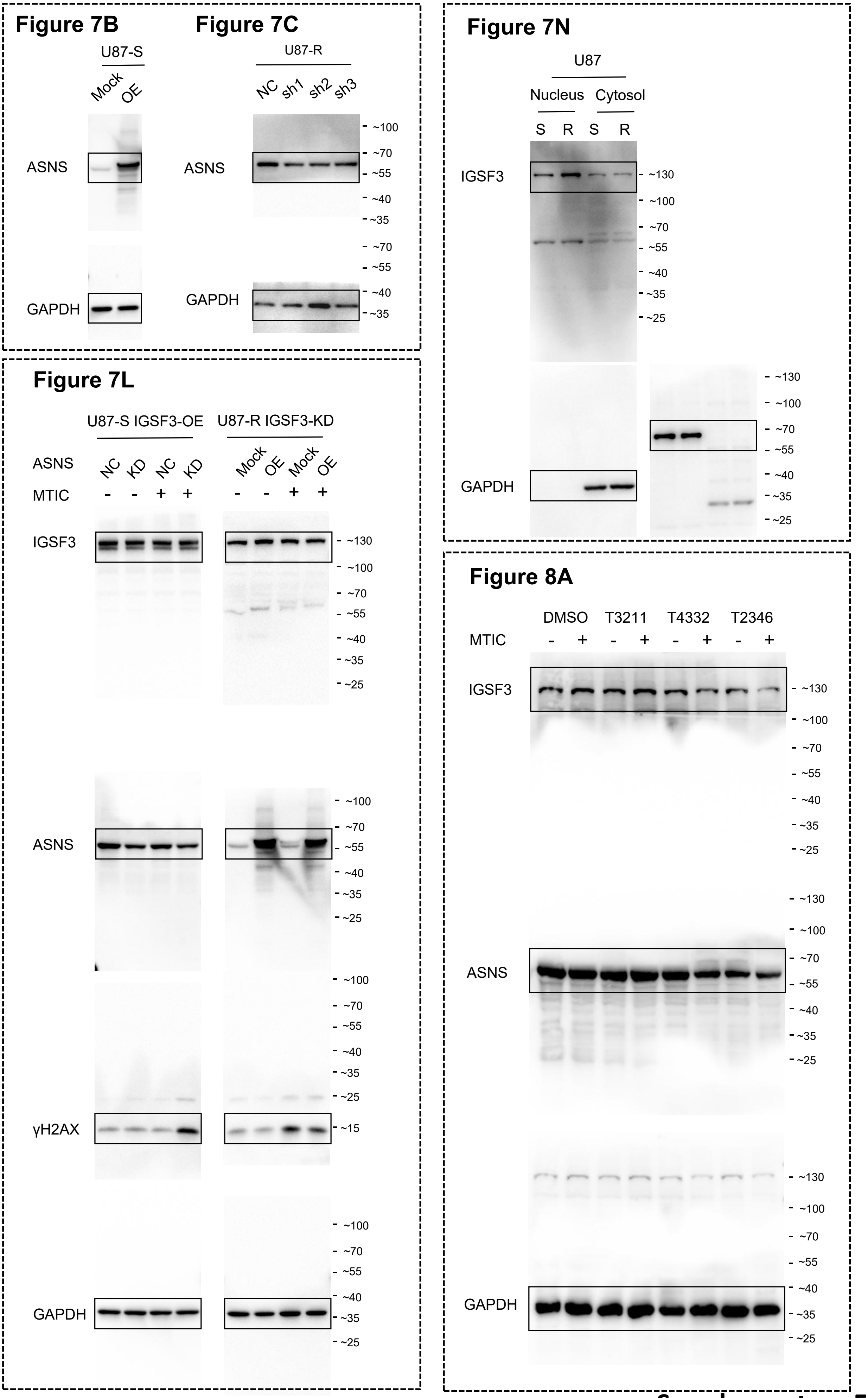

Supplementary Figure 4

Figure 8C

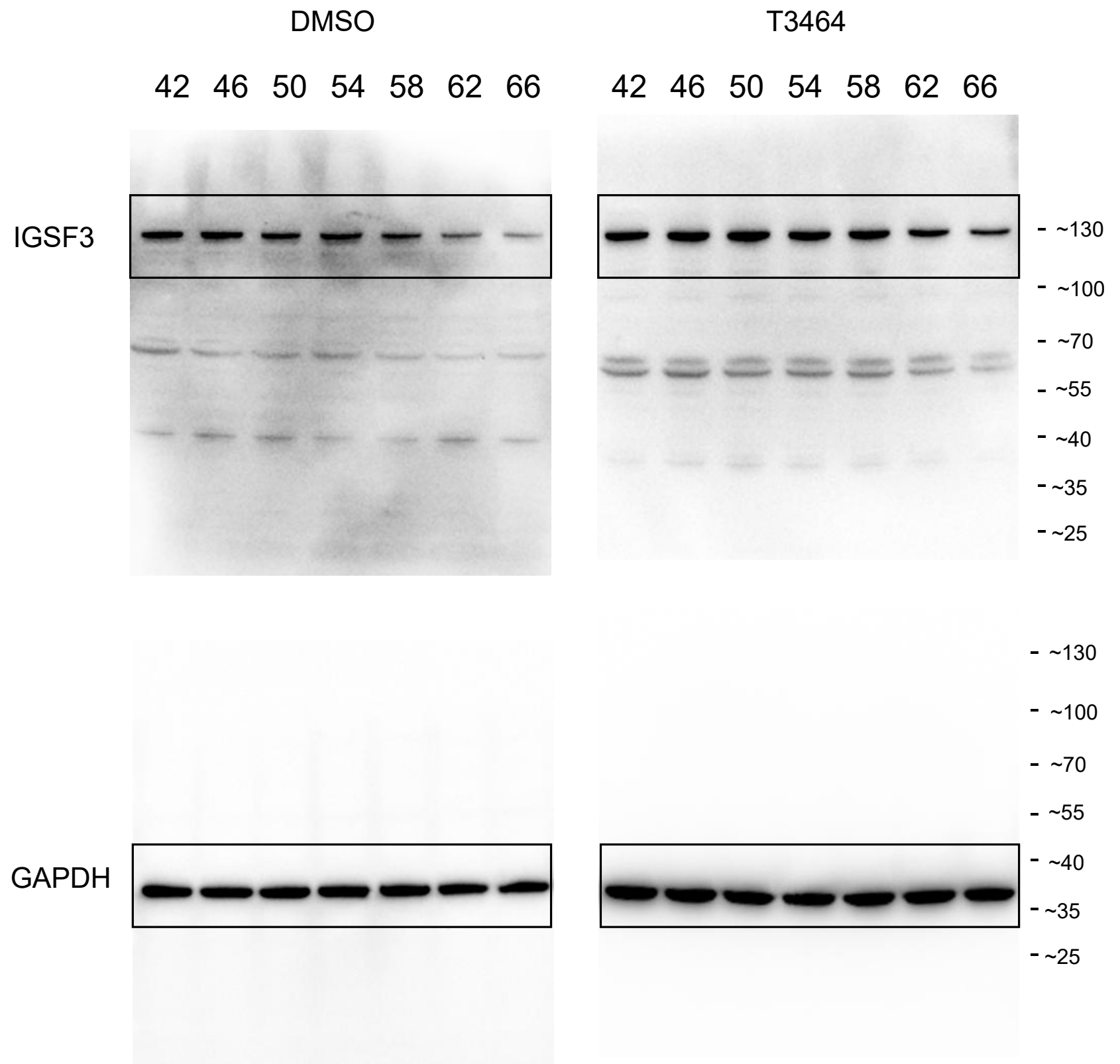

Supplemental Figure 3E

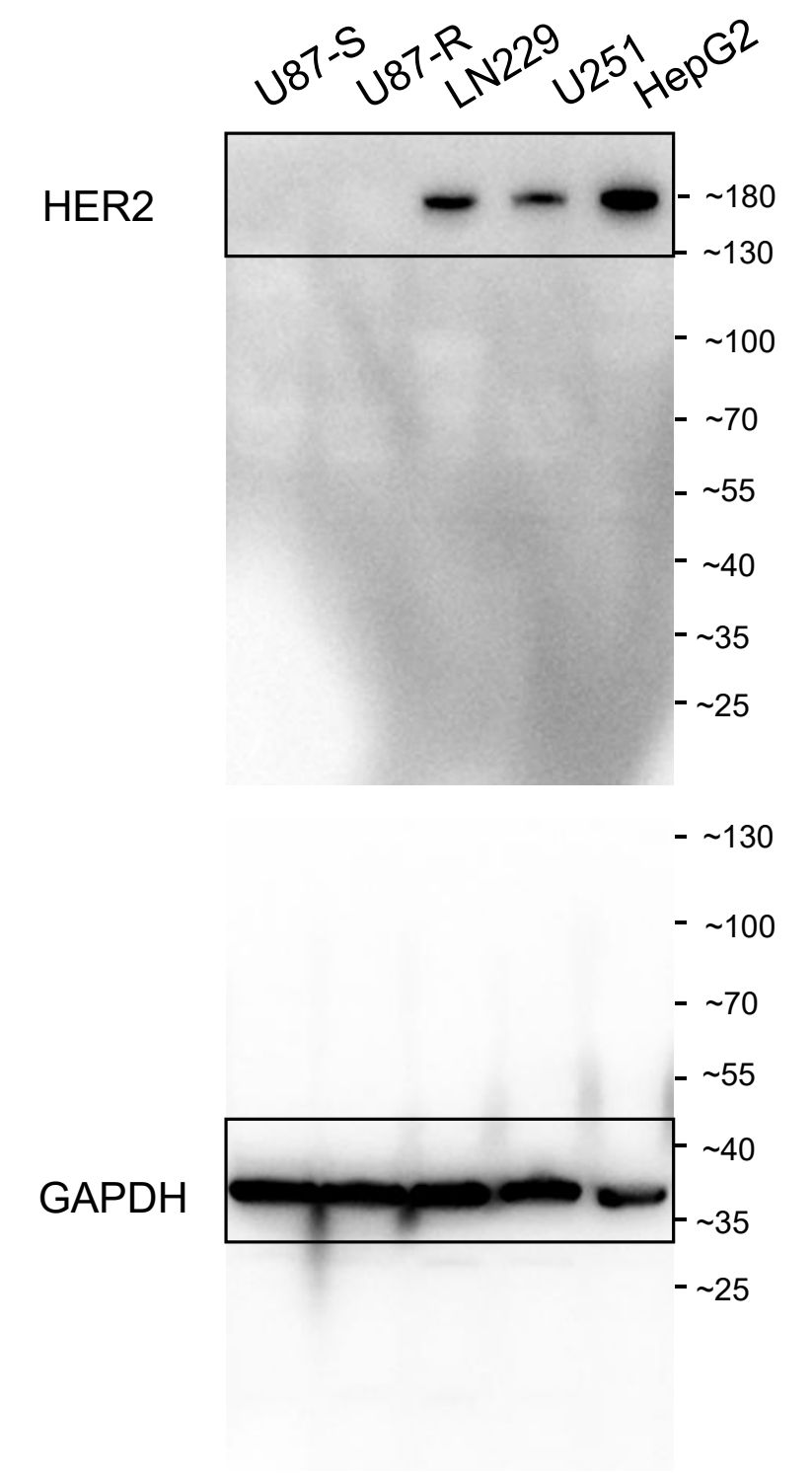

Figure 8G

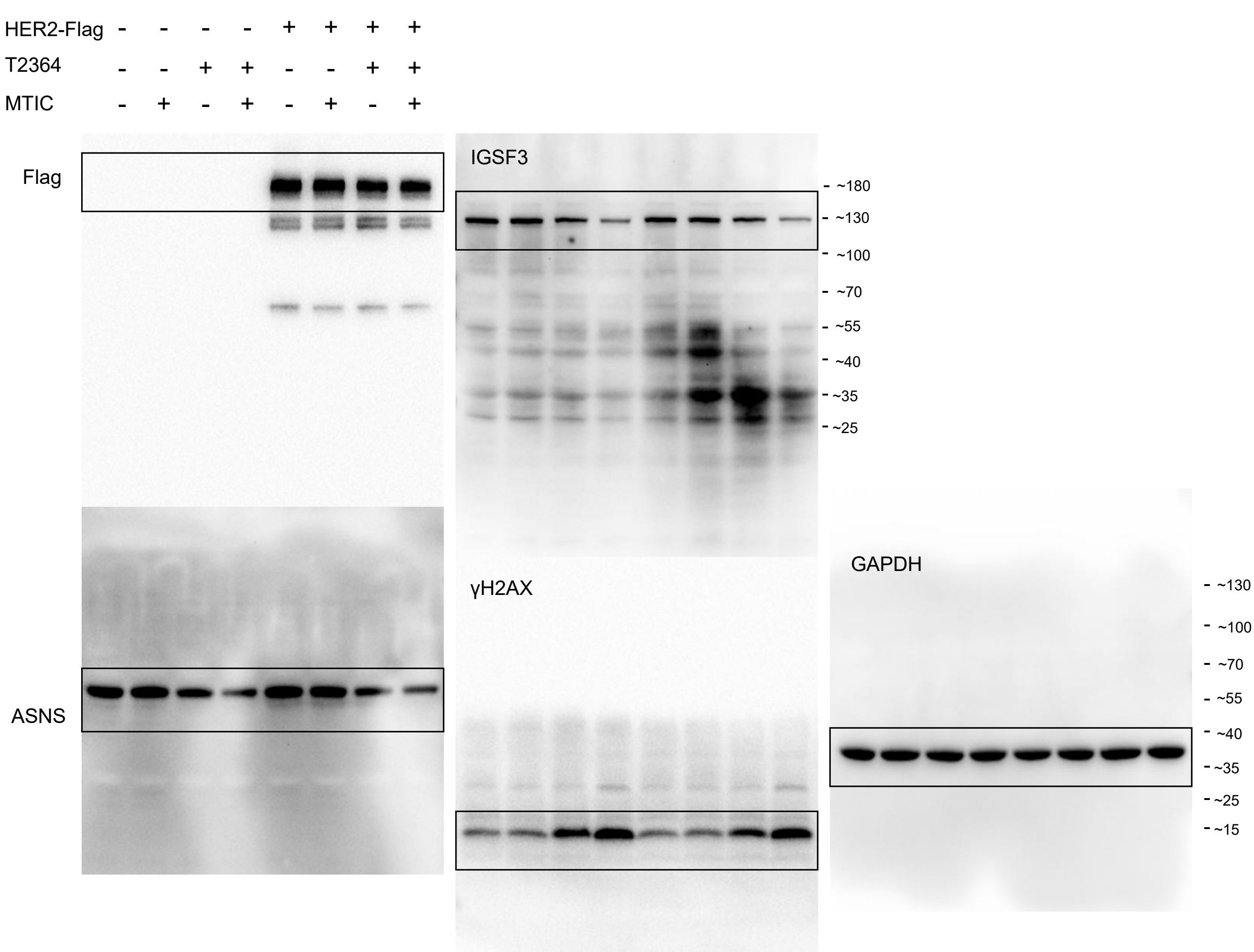

Supplementary Figure 4
